# Multi-scale multi-level marine spatial planning: A novel methodological approach applied in South Africa

**DOI:** 10.1371/journal.pone.0192582

**Published:** 2018-07-03

**Authors:** Erwann Lagabrielle, Amanda T. Lombard, Jean M. Harris, Tamsyn-Claire Livingstone

**Affiliations:** 1 Institute for Coastal and Marine Research, Nelson Mandela University, Port Elizabeth, South Africa; 2 UMR ESPACE-DEV 228, Université de La Réunion, IRD, La Reunion, France; 3 Scientific Services, Ezemvelo KZN Wildlife, Pietermaritzburg, South Africa; 4 Wildlands Conservation Trust, Hilton, South Africa; Auburn University, UNITED STATES

## Abstract

This study proposes and discusses a multi-scale spatial planning method implemented simultaneously at local and national level to prioritize ecosystem management actions across landscapes and seascapes. Mismatches in scale between the occurrence of biodiversity patterns and ecological processes, and the size and nature of the human footprint, and the different levels and scope of governance, are a significant challenge in conservation planning. These scale mismatches are further confounded by data resolution disparities across and amongst the different scales. To address this challenge, we developed a multi-resolution scale-linked marine spatial planning method. We tested this approach in the development of a Conservation Plan for a significant portion of South Africa’s exclusive economic zone, adjacent to the east coast province of KwaZulu-Natal (the SeaPlan project). The study’s dataset integrated the geographic distribution of 390 biodiversity elements (species, habitats, and oceanographic processes) and 38 human activities. A multi-resolution system of planning unit layers (PUL), with individual PUs ranging in resolution from 0.2 to 10 km, was designed to arrange and analyse these data. Spatial priorities for conservation were selected incrementally at different scales, contributing conservation targets from the fine-, medium- and large-scale analyses, and from the coast to the offshore. Compared to a basic single-resolution scale-unlinked plan, our multi-resolution scale-linked method selects 6% less conservation area to achieve the same targets. Compared to a multi-resolution scale-unlinked plan, our method requires only an additional 5% area. Overall, this method reflects the multi-scale nature of marine social-ecological systems more realistically, is relatively simple and replicable, and serves to better connect fine-scale and large-scale spatial management policies. We discuss the impacts of this study on protected area expansion planning processes in South Africa. This study showcases a methodological advance that has the potential to impact marine spatial planning practices and policies.

## Introduction

Marine spatial planning (MSP) is a decision support process for integrated ocean governance and aims to allocate marine and coastal resources sustainably and efficiently through space and time, to achieve social, economic, strategic and ecological objectives [1]. Within MSP frameworks, the focus of conservation planning is to identify spatial priorities for the protection of natural assets, and to identify spatial contraints for human activities in order to promote the persistence of functional coastal and marine social-ecological systems [2–3]. A key challenge in developing marine spatial management plans that are implementable and lead to action, is the integration of different scales of ecological and social systems [4]. There is growing recognition that conservation planning concepts and tools that address cross-scale dimensions are needed [5] to account for the multiscalar nature of conservation problems and to balance divergent priorities at multiple spatial scales [6]. Spatial conservation planning exercises generally address fine- and large-scale domain in separate planning processes, and social frameworks (e.g. legal, institutional, political) are often poorly integrated across local, national, regional and global scales [7, 8]. Ecosystems, however, are increasingly threatened at all scales [8] whereas local actions can have combined and cumulative impacts at broader scale, and global drivers of change, such as climate-related drivers, affect social-ecological systems locally [9]. Therefore, to maintain adaptive social-ecological systems, societies need to address the challenge of fitting the scale of their conservation actions to the spatial and temporal scale of social-ecological processes [6, 10, 11].

The need for conservation planning to address cross-scale dimensions is widely acknowledged and methods to balance divergent priorities at multiple spatial scales have been proposed [5,7] but rarely implemented in practice [6]. As a result, spatial conservation plans often make recommendations that do not fit the scale of local management, and local decisions often fail to address broader scale issues [7]. Scale mismatches can significantly limit the applicability and implementation of spatial plans in the real and complex world [5, 6, 7]. This problem arises largely because conventional spatial planning methods typically channel all data into a single-scale framework, and methods that effectively address the multi-scale and multi-resolution nature of data are lacking. In the marine environment, the data resolution and scale mismatch is particularly problematic. It is therefore “timely to explore tools and approaches that can help deal with scale mismatches that impede effective implementation” [6]. More generally, the multi-scale nature of social-ecological systems has raised increasing scientific interest from various fields, including mathematics, astronomy, physics, ecology, economics and geography [12,13]. Scale research has progressed with the development of fractal theory that defines the concepts and metrics for the analysis of multi-scale phenomena within a single notional framework [14]. In the fields of ecology, landscape ecology and biogeography, the analysis of ecological patterns and processes across scales has always been a central problem [15,16] both in the terrestrial and marine realm [17–18]. An abundant literature describing the concepts, challenges and issues related to the multi-scale organisation of complex and coupled social, jurisdictional, political, cultural and ecological systems has emerged in the past thirty years [6, 8, 10].

In practice, regional and global MSP processes generally use large-scale data (large area coverage) with a low spatial resolution (10×10 km resolution and broader) whereas local or even national plans are generally based on fine-scale high-resolution data (e.g. 1×1 km resolution) (see [19–23]). The move towards multi-scale multi-resolution MSP is technically facilitated by the increasing availability of data for both social and ecological systems that spans diverse scales and resolutions in both space and time [24–26]. Indeed, spatial marine ecology data from remote sensing satellite and ecological niche models [27,28], oceanographic circulation models [29], transport simulation models [30] and tracking devices [31] have also become increasingly available, allowing the monitoring of biophysical oceanographic parameters and biota movement across entire oceans. In addition, geographic data on human uses of the sea (e.g. Automatic Identification System ship location data) and management zonation data have become available and accessible. Exploring this data and using it to support decision requires integrative multi-scale multi-resolution methods.

This study proposes and discusses a multi-scale spatial planning method implemented simultaneously at local and national level to prioritize ecosystem management actions across landscapes and seascapes. This approach is tested in the development of a Conservation Plan for a significant portion of South Africa’s exclusive economic zone, adjacent to the east coast province of KwaZulu-Natal (the SeaPlan project). The hypothesis defended in this research is that a multi-scale and multi-resolution approach to spatial planning from its early steps (e.g. problem assessment and formulation [8]) can improve the efficiency of the planning process at the later implementation stages. In this study, the scale refers to the size of a spatial extent and the resolution refers to the smallest identifiable element of a spatial dataset. We present a technical procedure that links the systematic conservation planning [32], spatial prioritisation process across different scales. Firstly, we describe the method (i.e., multi-resolution scale-linked (ML)), the stakeholder involvement strategy, the data collection, analysis and modelling, and the decision implementation process. Secondly, we assess the advantages of the ML approach by comparing the outputs of the ML method with that derived from conventional single-scale single-resolution methods [29]. Finally the impacts of the plan on policy and future methodological developments are discussed.

## Study area and project context

### Ecological system

**Fig 1:**
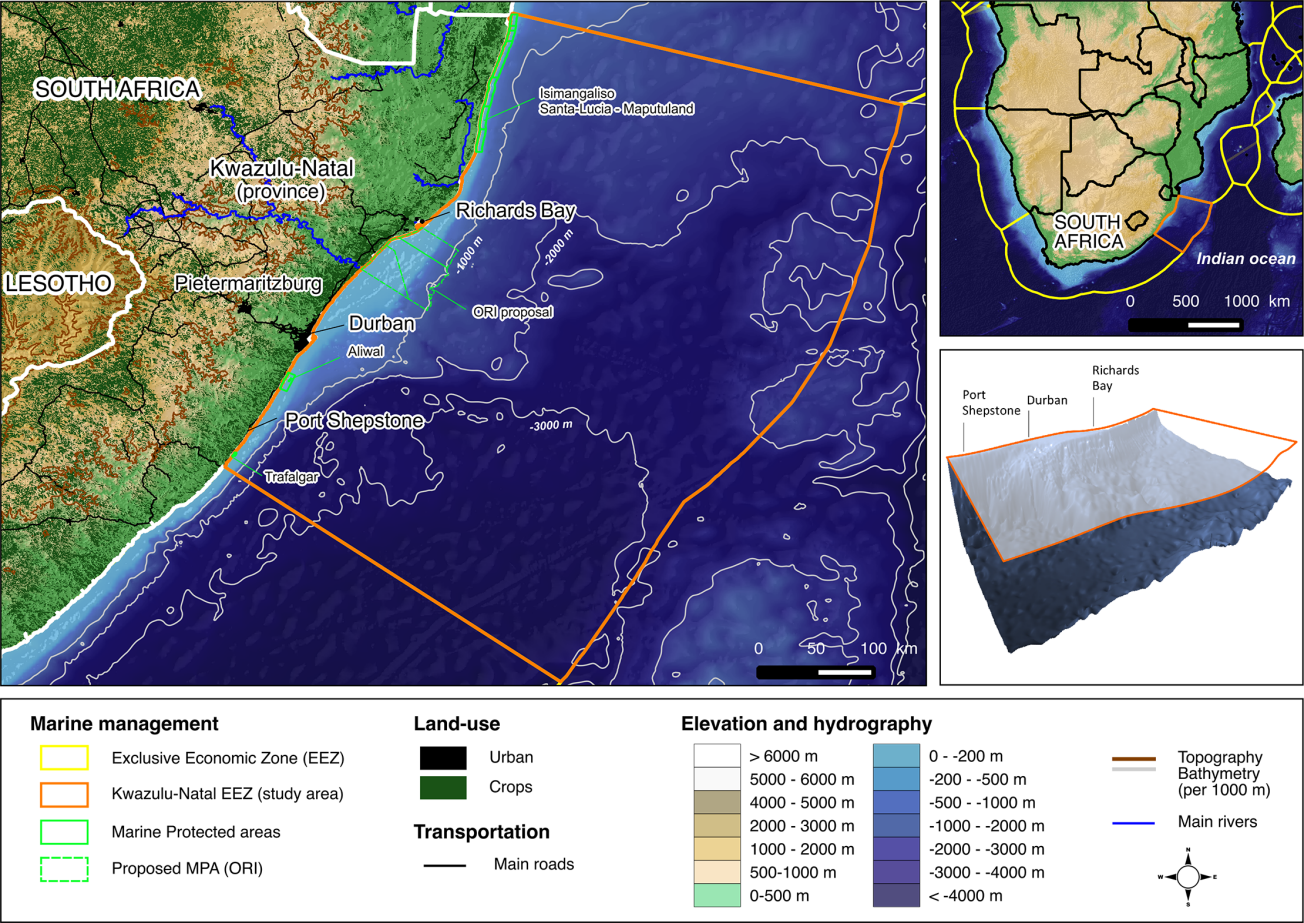
Study area map. Study area off the KwaZulu-Natal Province in the EEZ of South Africa, with a three-dimensional representation of the planning domain.

The study area is adjacent to the province of KwaZulu-Natal (KZN) on the east coast of South Africa, covering 640 km of coastline and 233,747 km^2^ of (EEZ) waters (Fig 1). The seafloor is characterised by a narrow continental shelf (<200 m) and steep continental slope, incised by canyons running down to the abyssal plain (maximum depth of -3,668 m). The offshore waters of KZN are dominated by the fast-flowing southward Agulhas current, which is largely formed by the recirculation of the South West Indian Ocean sub-gyre, with rings shed from the Mozambique and East Madagascar current [33]. The KZN waters are a typical oligotrophic system with low productivity. Owing to its strength and extent, the Agulhas current plays an important role in the distribution of species in the KZN region by transporting warm, salty tropical waters southward along the KZN coastline [33].

On the continental shelf, rocky reefs are distributed throughout the province, whereas the occurrence of coral reefs increases in the northern part of the study area [34]. Top predators such as tuna and seabirds concentrate in the more productive areas characterised by strong eddies [35]. The KZN marine environment consists of an estimated 1,640 marine fish species [36]. Coelacanths have been found in the northern canyons at depths of -54 to -359 m [37]. Loggerhead (*Caretta caretta*) and leatherback (*Dermochelys coriacea)* turtles nest along the northern stretches of KZN and 15 cetacean species have been found in KZN waters [38].

The coastline and nearshore have high habitat biodiversity, including rocky, sandy and mixed shores, rock platforms, mangroves and estuaries. The superficial coastal sediments consist mainly of sand with gravel and mud. Sediment inputs are mainly of fluvial origin [39]. Soft sediment habitats are often ephemeral owing to the effects of wave and tidal action on sand transport. These habitats are distributed throughout the province, and are typically inhabited by burrowing and benthic species [39].

The Province contains 74 river systems and associated estuaries, 13 of which are permanently open. The main river is the uThukela, contributing to more than 38% of the provincial freshwater flow. Estuaries form a vital connection between the different life cycle stages for many fish species. Some marine species spawn within estuarine systems and many breed at sea, with their juveniles using estuaries as nursery areas [40,41]. The largest mangrove areas in South Africa occur in northern KwaZulu-Natal estuaries, covering about 1391 ha [42].

### Social system and pressures on marine ecosytems

The human population in KZN has increased from 8.4 million inhabitants in 1996 to 10.5 million in 2013 (density of 110/km^2^) with 3.5 million located in the coastal Durban metropolitan areas [43]. The economy of KZN has been growing rapidly as a result of the post-apartheid political change and the integration of the South African economy within the global market economy [44]. In 2011, the average annual income per household per year was about 2,500 US dollars but 48.4% of the population still lives in poverty [43]. Agriculture (mostly sugar cane) and forestry remain central to the economy. Tourism accounted for 5.1% of the Gross Domestic Product in 2013 (IHS Global Insight database). In the marine environment, Durban and Richards Bay harbours are respectively one of the ten largest container terminals in the world and the area has largest coal export facility in Africa.

Offshore mining of heavy mineral sands, and increasing seismic activity for oil and gas industries, are placing coastal and marine ecosystems under increasing pressure. The cumulative threats to sandy beaches in South Africa are highest in the southern half of the KZN coastline [45]. Several marine fish stocks in KZN waters have declined or collapsed, with many no longer financially viable [46]. This trend has been exacerbated by new developments in artisanal fishing gear. International and local commercial fishing fleets target swordfish, sharks and tuna and are regarded as a potential threat to marine ecosystems. Commercial prawn trawling on the uThukela Banks has been shown to be severely detrimental to several fish and shark species owing to the industry’s high bycatch [47–49]. Shark nets pose a threat to many marine species, but particularly the larger non-target species such as turtles and cetaceans [50, 51], which are exposed to additional pressures including vessel collisions [52], plastic [53] and noise pollution [54].

Although fishing remains the greatest direct pressure on marine biodiversity, climate change has strong ecological, fisheries, resource management and socio-economic implications. In KZN, sea surface temperature (SST) has risen along the east coast [55] and is expected to result in the southward expansion of the ranges of tropical intertidal species, as has been recorded for some fish species [56]. Coral communities have experienced less bleaching than those in southern Mozambique, but the emerging trend is one of increasing frequency and intensity of bleaching [34]. Along the coastline, including the Durban metropolitan area, areas within 100 m of the shoreline are particularly vulnerable to sea-level rise and increased coastal erosion linked to an increased frequency and severity of storms [33].

### Current MPA network

As in the rest of the world, the establishment of MPAs in KZN has fallen far behind the proclamation of terrestrial protected areas [57, 58]. Nevertheless, the rapid increase in KZN’s coastal population and the mounting demands made on the marine environment led to the realisation of the need to establish a system of MPAs along the coast during the late 1960s [59]. KZN currently has three MPAs all of which occur inshore. In the North, covering the entire coastline of the Delagoa Bioregion [60], the marine component of the iSimangaliso Wetland Park (a UNESCO World Heritage Site) is divided into the St Lucia MPA (441 km^2^, proclaimed in 1979) and the Maputuland MPA (384 km^2^, proclaimed in 1986), both extending 3 M (nautical mile, 1 M = 1852 m) offshore. In the Natal Bioregion further South, there are two smaller MPAs: the Trafalgar MPA (proclaimed in 1979) is only 8.1 km^2^, 4.5 km long and extends 1 M offshore, and the Aliwal Shoal MPA (proclaimed in 2004) is 125 km^2^, 18 km long and extends to 4 M offshore. All four of the above mentioned MPA’s have been proclaimed (or re-proclaimed) under Section 43 of the Marine Living Resources Act (Act 18 of 1998) and the two MPAs within the iSimangaliso Wetland Park have also been proclaimed under the World Heritage Convention Act (Act 49 of 1999). It is important to note that the Pondoland MPA (currently South Africa’s largest continental MPA in terms of surface area ~900 km^2^) was also proclaimed in June 2004 just south of the KZN/Eastern Cape border. Also of relevance is the recent proclamation of the Ponta do Ouro Partial Marine Reserve in Mozambique extending northwards from the RSA/Mozambique border to inland of Inhaca Island.

### The SeaPlan marine spatial planning project

In the late 1990s, Ezemvelo KZNWildife (EKZNW), the conservation authority for South Africa’s Kwazulu-Natal (KZN) Province, initiated the SeaPlan marine conservation planning project [61], to be implemented along the 640 km coastline of KZN and its Provincial waters, including all marine habitats (coastal, estuarine, benthic and pelagic), from the shoreline out to the offshore limits of the EEZ. EKZNW has the institutional mandate to implement marine conservation actions, to facilitate stakeholder participation, and to recommend plans for resource use and protection of biodiversity. EKZNW is contracted by the national environmental agency to manage proclaimed MPAs in the province of KZN.

SeaPlan aimed to 1) assess the status of marine biodiversity protection in the province; and 2) identify spatial priorities for future marine conservation actions. The overarching challenge was to achieve these objectives across spatial scales and levels of governance, while engaging meaningfully with stakeholders from many sectors of society. As the first marine spatial planning process in South Africa, SeaPlan also aimed to contribute methodological guidelines toward the integration of biodiversity conservation priorities with other MSP processes at Provincial and National levels, for example, the National Protected Area Expansion Strategy [62] and more recently, the “Marine Protection and Governance” component of the National Phakisa Operation (http://www.operationphakisa.gov.za) which aims to promote a sustainable ocean economy through MSP. The need for an expansion of the MPA estate in the Natal Bioregion of KZN, and particularly its offshore areas, is recognized both Nationally and Provincially. Although the entire Delagoa Bioregion in the North falls within a protected area (Fig 1), only 6% of the Natal Bioregion falls in a MPA and only 0.28% of offshore areas are protected. In addition, the Natal Bioregion MPAs do not include any fully protected (or “no-take”) sanctuary zones.

The SeaPlan development process follows adaptive management principles. Its host (EKZNW) has the research capacity to collaborate, gather and analyse data, respond to opportunities and constraints, engage with stakeholders, update plans, and make recommendations to either local or national government for MPA expansion, in an on-going process which is part of its mandate [23]. South Africa’s national marine planning products [63,64] provide the most complete MSP study for the EEZ, but do not integrate inshore and offshore planning. SeaPlan is currently the only study that spans the coastal-inshore-offshore continuum, making use of all available data for both biodiversity and human use. The current iteration of SeaPlan aimed to develop a multi-scale multi-resolution conservation assessment and plan for the next 20 years. Here we present the methodology developed to achieve this objective.

## Material and methods

### Overview

**Fig 2:**
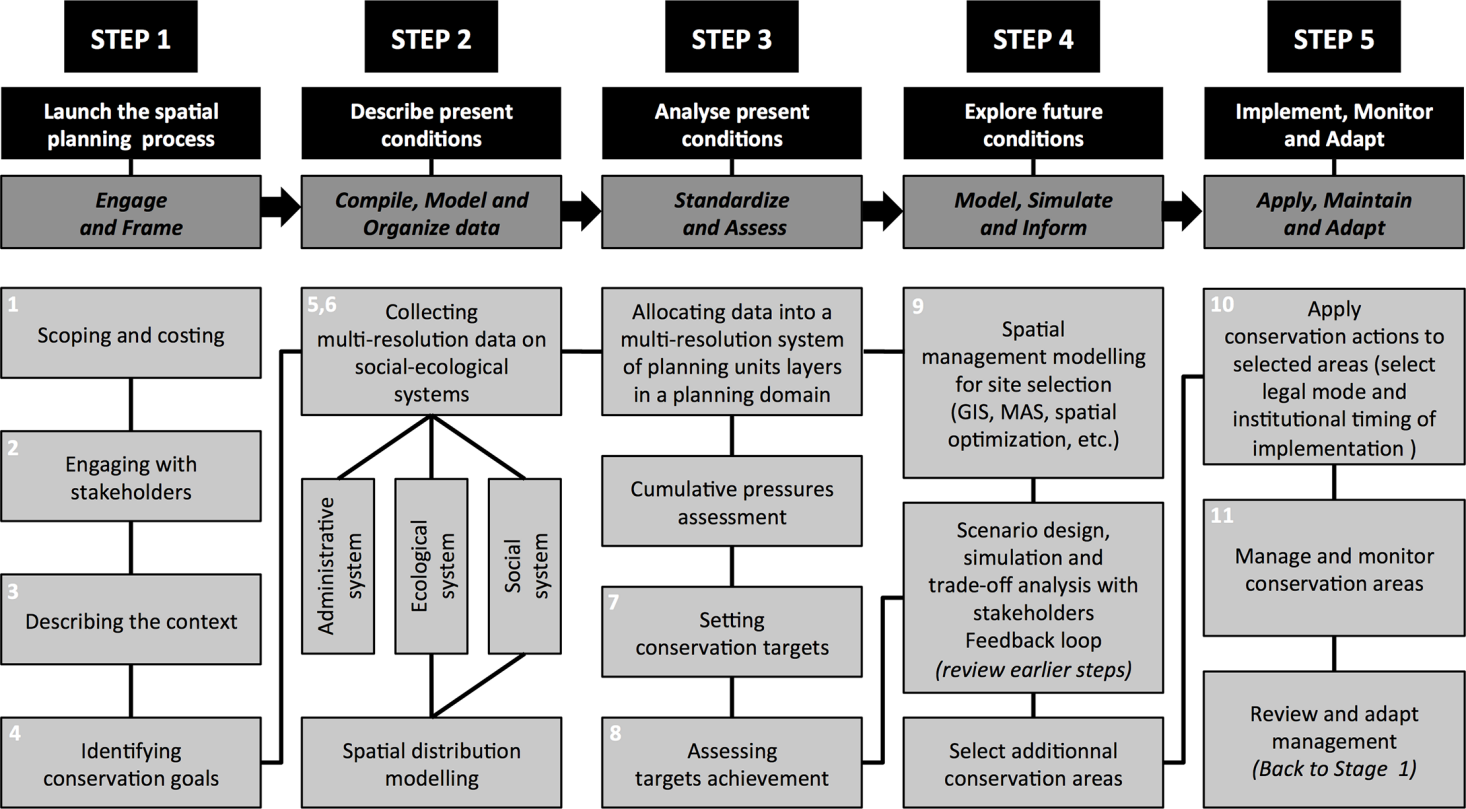
Key steps within the multi-scale marine spatial planning (MSP) process. Launch the MSP process (step 1), Describe present conditions (step 2), Analyse present conditions (step 3), Explore future conditions (step 4) and Implement, monitor and adapt (Step 5). Generic steps were adapted and expanded from [22] and [24]. Number in white refer to the eleven-stages of Systematic Conservation Planning by [25] expanded from [32].

SeaPlan used a five-step method to identify and implement future priority areas for conservation action across different scales in the study area. These steps follow the systematic conservation planning framework [22, 24, 25, 32]. The steps are summarised in Fig 2, and then detailed in following sections. Step 1 was initiated by EKZNW in the late 1990s, involving multi-level decision-makers and stakeholders operating or managing coastal and offshore activities. Step 2 collected or collated spatial distribution data on biodiversity (species, habitats, oceanographic processes) and human activities in the marine environment: 85 marine species, 264 habitats (shoreline, benthic and pelagic) and 38 human activities (presence/absence, with intensity gradients for 15 of them). At least ten years were required to assemble these data sets. Step 3 developed an assemblage of three spatially nested planning unit layers (PUL): a fine-scale high-resolution PUL (0.2×0.2 km), a medium-scale medium-resolution PUL (1×1km), and a large-scale low-resolution PUL (10×10 km). Data layers from Step 1 were allocated to one of the three PULs, in what we refer to as a “scale allocation” process. Conservation targets (quantitative expressions of conservation goals) were set to specify how much of each biodiversity element (species, habitat types, oceanographic processes) should be protected. Targets were set as a percentage of the distribution area of each element, and the target achievement within the current MPA network was assessed. In Step 4 an efficient and practical spatial arrangement of additional multi-resolution PUs was identified to meet all (currently unmet) targets in the entire EEZ. The final network of MPAs maximised target achievement within sites that were spatially clustered at all scales (i.e. fine, medium, large) while minimizing overlaps with other human activities. Step 5 entails the implementation, monitoring and adaptation of the conservation actions planned in selected PUs. This final step underway is described in the Discussion section. All analysis were conducted using ArcGIS 10 (Environmental Systems Research Institute, Redlands, California).

### Step 1: Launch the spatial planning process

The objective was to develop a multi-scale vision for the marine spatial planning process through cross-sectoral engagement with stakeholders. This step encompasses the first four SCP steps [22, 24, 25, 32], i.e. scoping and costing the planning process; identifying and involving stakeholders; describing the context for conservation areas; and identifying conservation goals. To initiate the process, individual interviews with specialist scientists and small workshops (less than 20 participants) with relevant user groups were held to identify, collect or build spatial datasets on ecological features and human activities, for example, coral reef distribution, rocky reef location and shore angling effort. This initial step identified realistic expectations for the conservation plan and assessed the legal mandates and political capacities of the implementing institution.

Public participation was encouraged to set up a collective vision and translate it into measurable objectives. Since its inception, SeaPlan has directly involved over one hundred stakeholders operating at different scales in the marine and coastal domain, including the commercial fishing sector, the recreational sector (e.g. fishers, scuba divers, surfers, etc.), conservation scientists, MPA managers, conservation non-government organisations (NGOs), urban planners, and other government sectors. Participants were kept informed of the spatial planning steps and preliminary products through regular emails and involvement in collaborative workshops. A larger annual workshop was held each year during the planning process from 2009 to 2012. In 2009, the multi-scale conservation assessment results and the site prioritisation method were presented at a a two-day public workshop to over 100 participants. In 2010, the outputs of the spatial prioritisation process were presented and discussed with a core group of 50 multi-sectoral stakeholders, MPA managers and scientists. Preliminary maps of proposed MPAs were presented during this meeting, and the outputs were adjusted to accommodate additional information and stakeholder interests. In 2011 a meeting was held to discuss final changes to the conservation plan and set of proposed new MPAs (50 participants). In 2012, a final stakeholder workshop was held to discuss processes for future updates to the conservation plan and strategies to implement a prioritised subset of selected proposed MPAs (50 participants).

### Step 2: Describe present conditions

#### Collection of data on social and ecological systems

Individual interviews with specialist scientists and small workshops (less than 20 participants) with relevant user groups were held to identify, collect or build spatial datasets on ecological elements and human activities, for example, coral reef distribution, rocky reef location and shore angling effort. Various types of spatial data were collected or collated at different scales on the administrative, ecological and social systems. Administrative data describe the spatial distribution of management regimes in the seascape (MPAs for instance). Ecological data described habitats (water masses, oceanographic processes, shoreline habitats, coral reefs, rocky reefs, submarine canyons and estuaries) and species distribution (fish, turtles and cetacean) (S1 Appendix). Data on social system focused mostly on human uses (mapping of activities) (S2 Appendix).

Existing and proposed MPA boundaries were mapped. All these protected areas comprise coastal and marine areas between the vegetation line and the limit of the EEZ. Existing MPA boundaries were extracted from the South African Government Gazette (Marine Living Resources Act). Spatial data were collected for other protected areas that overlapped marginally with the planning domain: terrestrial protected areas (data from EKZNW), and Admiralty reserves and State Land (data provided by the KZN Provincial Planning & Development Commission). The boundaries of six proposed MPAs were mapped with MPA proposers.

#### Modelling spatial distributions of biodiversity elements and human activities

A range of ecological modelling techniques and tools were used to model the distribution of habitats and species (time series analysis, cluster analysis, boolean rules, etc.). S1 Appendix describes the ecological data that were collected or modelled (biodiversity conservation elements). S2 Appendix describe the method for mapping the presence/absence or the intensity of 38 human activities that can impact marine ecosystem structure and function negatively.

### Step 3: Analyse present conditions

#### Planning domain delineation

**Fig 3:**
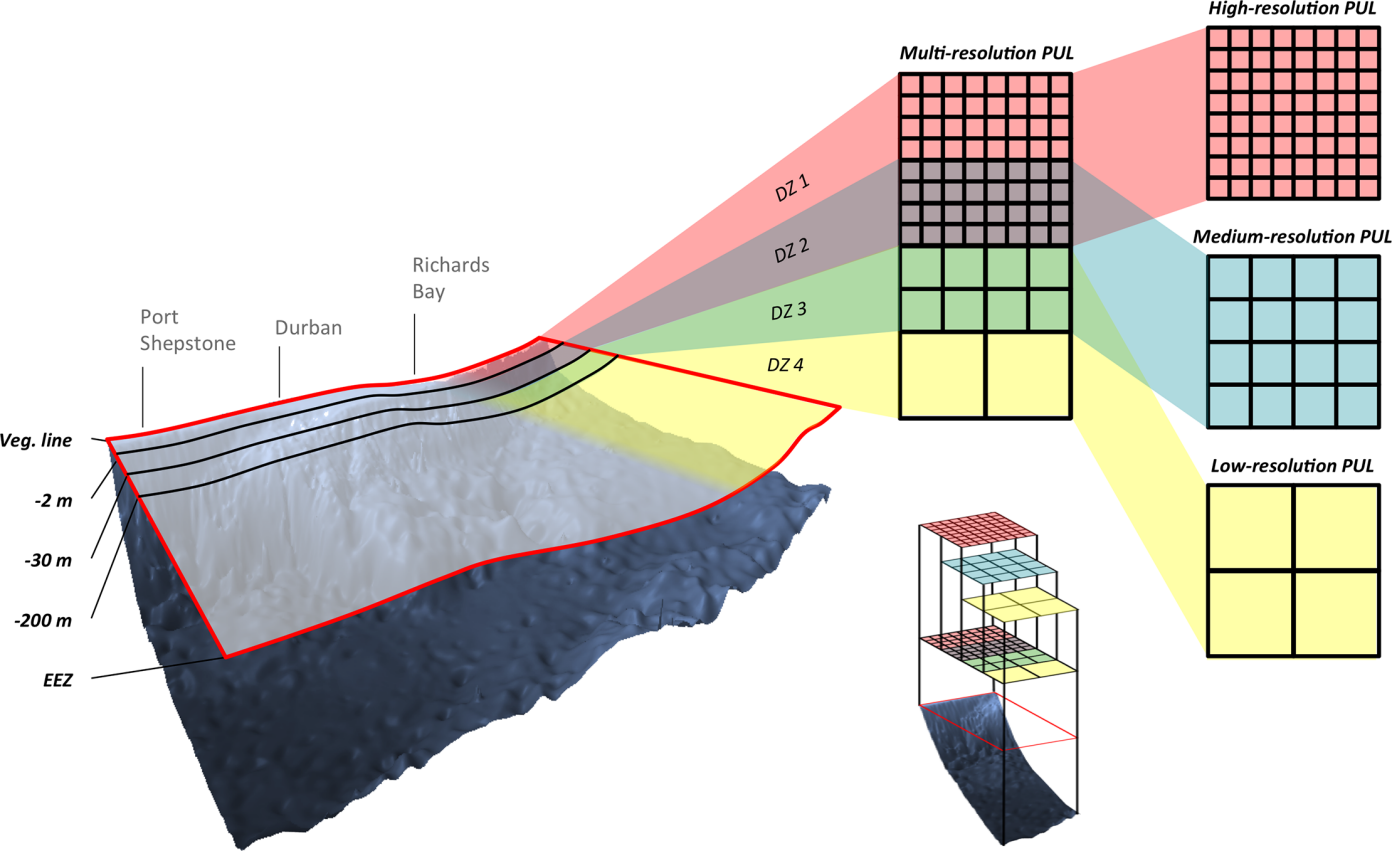
Multi-resolution nested planning units layers. The planning domain is divided into fours depth zones (DZ) and are covered by three partially overlapping multi-resolution planning unit layers. The high-resolution planning units layers (red) covers DZ 1 and 2 (vegetation line to -30 m). The medium-resolution planning units layers (blue) covers DZ 2 and 3 (-2m to -200 m). The low-resolution planning units layers (yellow) covers DZ 3 and 4 (-30 m to the limits of the Economic Exclusive Zone). “Veg. line” stands for vegetation line and “EEZ” for Economic Exclusive Zone.

The planning domain stretched from the coastal vegetation line, delineated from aerial photography, to the EEZ (Maritime Boundaries Geodatabase [65]). The planning domain was first divided into four depth zones (DZ): DZ one (vegetation line to -2 m depth), DZ two (-2 m to -30 m), DZ three (-30 m to -200 m) and DZ four (-200 m and deeper) (Fig 3). The -30 m and -200 m depth cuts were based on the following rationale: -30 m is the offshore limit of the deep photic zone in this region, where the influence of waves and light attenuates [49], and very little macroalgae occurs below 30 m. Additional biological rationale for this depth cut is that the sardine run occurs to this depth, and coral reefs occur up to about 30 m (although non-reef corals do occur in deeper subtidal habitats). The 200 m depth is the approximate offshore limit of the continental shelf in the Province.

#### Multi-resolution planning units layers

Three PULs were generated with squares at three different resolutions (Fig 3): the high-resolution PUL (0.2×0.2 km) covered DZ one and two, the medium-resolution PUL (1×1 km) covered DZ two and three, and the low-resolution- PUL (10×10 km) covered DZ three and four. Each of the PULs was then intersected with the boundaries of the existing network of conservation areas. This network included: MPAs (with internal zonation as described below), terrestrial protected areas, admiralty zones and state land. Depth contours were extracted from the bathymetry layer described previously. PUs smaller than 5,000 m2 were then dissolved into adjacent polygons, but keeping MPA boundaries, depth zone boundaries and the planning domain boundary intact. In addition, the three PULs were combined to form a unique “mixed-resolution” large-scale PUL required to perform the conservation assessment. The highest resolution PU taking precedence where there was overlap.This mixed-resolution PUL contained 64,189 planning units. It was not used in the site priorisation process that articulates three distinct PULs (fine-, medium- and large-scale PULs).

#### Allocating data to multi-resolution planning unit layers

**Fig 4:**
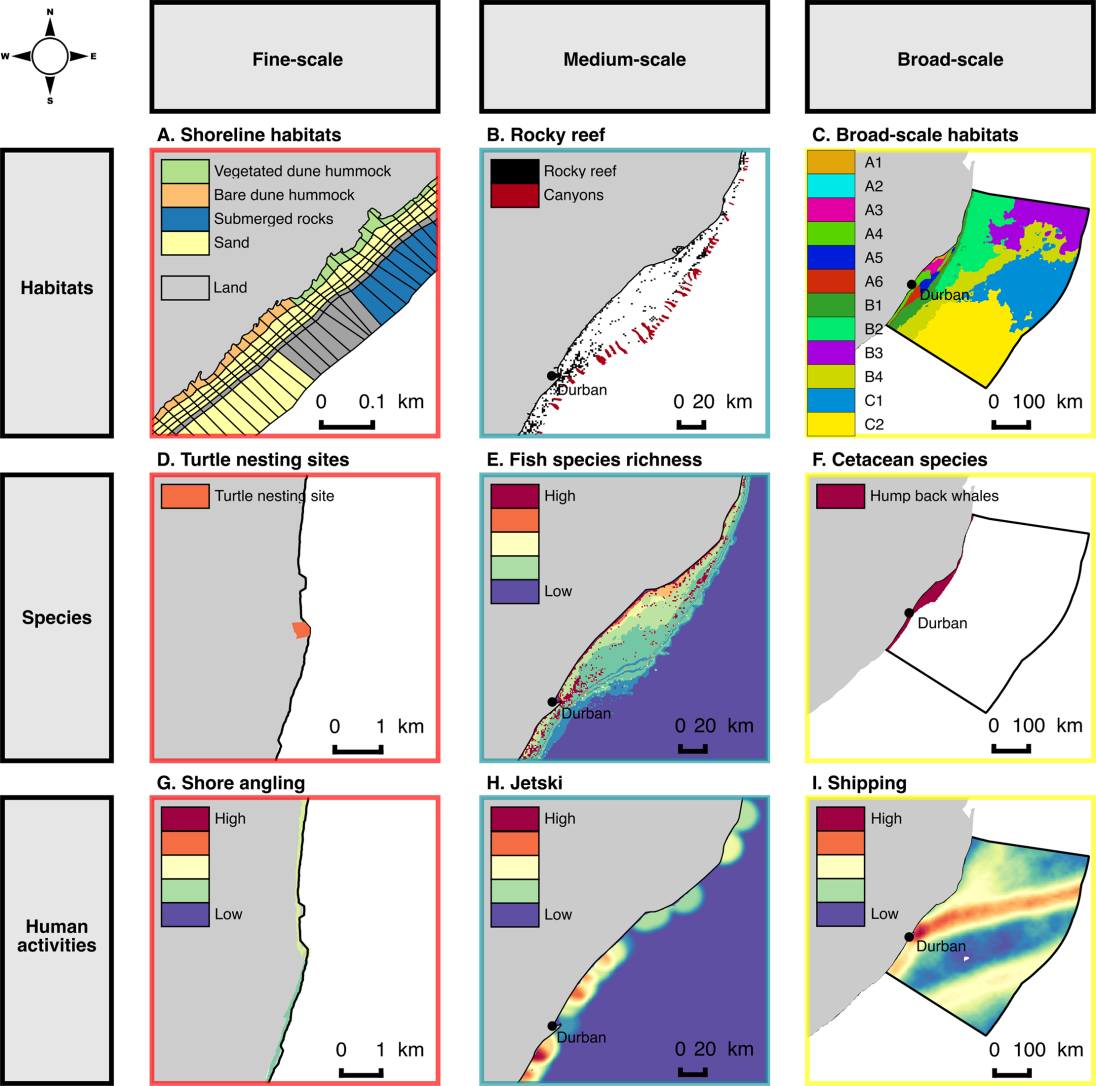
Example of multi-resolution spatial data. Maps showing (from A to I) extracts of the multi-resolution spatial distribution data for habitats, species and human uses at fine-, medium- and large-scale. Colors of map frames indicate the high-resolution planning units layers (red), the medium-resolution planning units layers (blue) and the low-resolution planning units layers.

Data were verified and converted into raster or vector layers in ArcGIS. Spatial layers for each biodiversity element were overlayed with the PU layer of the appropriate resolution and aerial coverage (Fig 4). Thus, the 0.2 km PUL was overlaid with fine-scale data along and close to the shoreline (e.g. the shoreline habitat data); the 1 km PUL with medium-scale data on the shelf (e.g. reefs), and the 10 km PUL with large-scale data offshore (e.g. oceanographic eddy distribution data). This “scale allocation” of data to PUs of appropriate resolution was a key step in the structuring of our multi-scale planning process. The mean cost value from the cost map was calculated for each PU at all three levels of resolution, based on the assumption that pressures are cumulative and can potentially affect marine ecosystems at all operational scales.

#### Assessing cumulative anthropogenic pressures

Data on the spatial extent and intensity of drivers of change (i.e. human activities) were combined into an index of cumulative anthropogenic pressure, which used an expert-driven weighting approach to assign higher values to activities with known high impacts (e.g. trawling), and lower values to activities with lower impacts (e.g. scuba diving) (see S2 Appendix). This method assumes that high cost values are a proxy for actual impacts, as well for areas where high socio-economic costs would be incurred in the event of conservation management measures being instituted. The weight was attributed to each of the 38 individual human activities (weights from 5 to 21.5) by a panel of experts according to their relative assumed impact on marine ecosystems. Layers were then weight-summed to create a single cumulative anthropogenic pressures map (with values linearly scaled from 0 to 100) that was linked to the main conservation elements (species, habitats and oceanographic processes) and latter used as a cost map in the priority sites selection procedure.

#### Setting and assessing conservation targets

Conservation targets were set under the authority of Ezemvelo KZNWildife (EKZNW), the conservation authority for South Africa’s Kwazulu-Natal (KZN) Province. Targets were set to comply with the objectives of the Convention of Biological Diversity (CBD), using IUCN status and through discussions with internationally recognized south african experts. Stakeholders validated conservation targets during SeaPlan public workshops but did not contribute to build them.

The Aichi-CBD targets state that by 2020, at least 10 per cent of coastal and marine areas should be conserved through effectively and equitably managed, ecologically representative and well-connected systems of protected areas and other effective area-based conservation measures, and integrated into the wider seascapes. Previous international policy statements issued by the World Summit on Sustainable Development (2002) and the World Parks Congress (2004) set a target for governments to protect 20–30% of all marine habitats under their jurisdiction (i.e. including Exclusive Economic Zones). In order to minimize the risk of a fishery collapsing owing to overexploitation, and recruitment overfishing, early calculations suggested that at least 20% of the spawner biomass of the unexploited stock is required to ensure sustainability [66]. This does not necessarily directly translate into a spatial requirement but has been used as the basis on which the World Parks Conference recommended that at least 20% of the waters within a country’s jurisdiction be protected [67]. Consequently, a baseline target of 20% was used for most of our conservation planning analyses.

The initial status of PUs was referred to as “available” or “conserved”. Within KZN, MPAs (or zones within MPAs) are classified as either Types A, B or C. There is currently no National standard for MPA zonation, but within KZN a consistent zonation is applied across the 3 existing MPAs. Type A MPAs are similar to no-take sanctuary areas with no extraction allowed, Type B MPAs allow restricted extraction activities that are compatible with the objectives of the MPA, and Type C MPAs allow additional regulated human uses. Note that only Types A and B were considered to contribute to meeting conservation targets within SeaPlan, because Type C MPAs currently do not provide any more biodiversity protection than areas outside MPAs.

A baseline target of 20% of a biodiversity element’s distribution area was applied to all elements. This baseline target was adjusted for some elements (by adding an additional 0–80%) based on their rarity, endemism, specialisation, localised distribution, and intrinsic vulnerability, as advised by experts. Final targets for each element had to be met equally within Types A and B MPAs (i.e. half the target in each Type) unless otherwise stated by experts (S3 Appendix). The existing MPA network in the study area was overlaid on all biodiversity elements to determine how much of A and B targets for each element were already met in Types A or B MPAs. Then, for each biodiversity element, the proportion of target achieved/unachieved was calculated.

Targets for habitats were fixed at 20% for large-scale marine habitats, rock reefs and fine-scale shoreline habitats (except for foredune habitats, surf-zone habitats, and submerged and emerged rocks which had 10% targets owing to the fact that they are common and widely distributed and considered more resilient than the other habitats). Coral reef targets ranged from 80% to 100% owing to their uniqueness, low resilience, high vulnerability and exposure to direct and indirect threats in the KZN region [30]. Canyon targets were set as follows in each bioregion (Natal and Delagoa): one canyon with 100% Type A protection and another with 100% Type B protection. Canyons were manually selected based on their proximity to existing MPAs. Targets for the modelled marine influence of 17 estuaries (of a total of 74) were set to 20% (of their area) within Type B protection (both estuaries in the Delagoa bioregion, and 15 of the 72 estuaries in the Natal bioregion). These 17 estuaries were identified for inclusion in SeaPlan by the Estuaries Spatial Conservation Plan [68], based on their irreplaceability value. Baseline targets for fish and cetacean species were fixed at 20%, with an adjustment target for each species based on the following criteria (additional 0–10% per criterion): endemicity, rarity, specialisation, localised distribution and intrinsic vulnerability [28]. Targets for areas supporting semi-permanent oceanographic processes (zones with frequent fronts or gyres) were set to only 10% of their distribution, but given that these areas are important for pelagic ecosystems and are species aggregation sites, we assigned the full target to Type A MPAs. The target for turtle nesting sites was set to 80% divided into 20% for Type A and 60% for Type B protection.

### Step 4: Explore future scenarios and solutions

#### Decision support system

We used MARXAN software [69] to identify networks of additional protected areas (i.e. reserves) that best achieved conservation targets. MARXAN was specifically designed to compute combination calculations for complex site priorisation problems. The software aims to solve the “minimum set reserve design” problem [69]. In this study, the use of Marxan contributed to ensure an unbiased and controlled selection of planning units along the multi-scale spatial planning procedures. Nevertheless, the multi-scale method exposed remains independant from the use of Marxan or any other spatial optimisation software. MARXAN algorithm uses a mathematical approach called simulated annealing [70] to identify near-optimal reserves portfolios (i.e. networks of protected areas) that minimize a cost function [71]. The cost function in MARXAN is the sum of an overall penalty factor (a cost is allocated if a conservation target remains unachieved) with an overall planning units cost. A planning unit cost can be a measure of any of its aspects, such as its area or perimeter, the probability of being impacted by human activities, or the opportunity cost resulting from its loss [68]. Each run of MARXAN produces a near-optimal reserve portfolio named solution. MARXAN produces two outputs: the “best solution” (i.e. the less costly solution calculated along the runs) and the “summed solution” (i.e. the cumulated number of selections of planning units as part of a solution along the runs). The number of runs and the calculation of costs can be parameterized heuristically by the user (see [72] for more details on MARXAN good practices). In our study MARXAN parameters were tuned with members of the Scientific Services unit of Ezemvelo KZN Wildlife.

#### Multi-resolution site selection procedure

**Fig 5:**
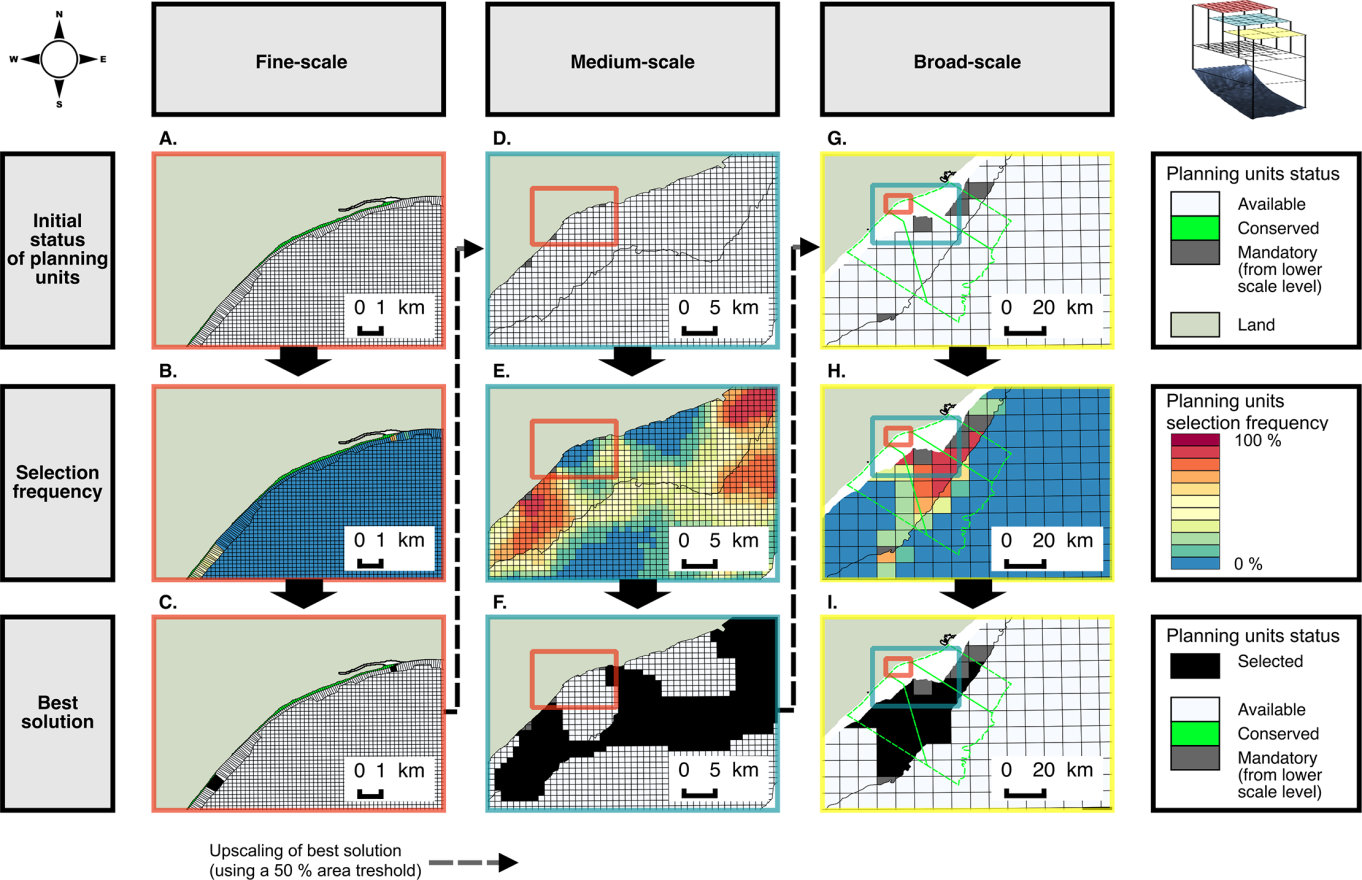
The multi-resolution site selection procedure to develop and coordinate the fine-, medium- and large-scale conservation plans. Maps showing the planning units status (A, D, G), the selection frequency of planning units (B, E, H) from MARXAN runs and the ‘best solution’ identified (C, F, I). Broader-scale planning units inherit a mandatory protection status if ≥ 50% of their area is selected as part of the “Best solution” at finer scale. Colors of map frames and map extent indicate the high-resolution planning units layers (red), the medium-resolution planning units layers (blue) and the low-resolution planning units layers (yellow). Dotted green lines indicate the MPA proposal made by ORI.

A bottom up site selection procedure was designed to identify conservation areas incrementally from the high-resolution PUL to the low-resolution PUL. In this procedure a PUL inherits mandatory planning units from the lower scale PUL. First, Marxan was run in the high-resolution PUL to select additional conservation areas that achieve targets for the fine scale biodiversity elements (Fig 5). Next, we ran Marxan on the medium scale elements with the medium resolution PUL (Run 2), keeping the mandatory sites from Run 1. Mandatory sites in Run 2 included MPAs and planning unit overlapping (>50% treshold) with the best solution identified in Run 1. The process was repeated for large-scale elements with the low-resolution PUL (Run 3).

The fine scale plan was run first because there are more constraints in the coastal and shallow areas given the proximity effects of increased human activity, whereas the large-scale plan encompasses the pelagic domain where more management options are available for conservation. Runs 1, 2 and 3 were performed to meet A targets and a second time to meet B targets (where the “best solution” for meeting A targets was retained, to ensure its inclusion in the final solution). The results of the fine, medium and large-scale plans were combined into the single multi-resolution PUL reflecting the “selection frequency” and “best solution” results for all PUs. The conservation penalty factor (PF), cost per PU, and the boundary length modifier (BLM), all required by Marxan, were assigned as follows: the PF was set very high (10 million per biodiversity element) to ensure that all conservation targets were met; the cost of each PU used the cumulative anthropogenic pressures map as previously described; and the BLM varied in relation to the PU resolution. A high BLM promotes compactness in the final selection of PUs. The BLM was set as follows for each PU layer (fine, medium, large scale): BLM = 100 × PU resolution (in metres).

### Comparing different methods of addressing multiple scales in spatial planning

**Fig 6:**
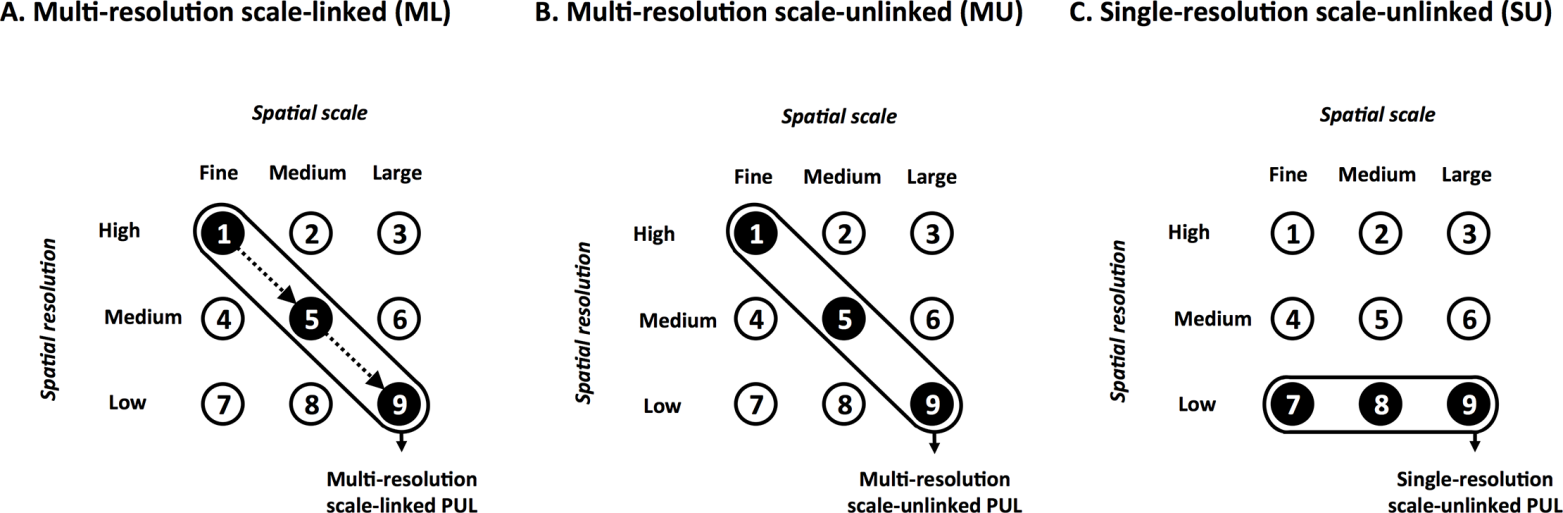
Three methods for addressing scale in spatial conservation planning. (A) The multi-resolution scale-linked (ML) planning method used for SeaPlan is described in this study (dashed lines arrows indicate inter-scale linkages), (B) The multi-resolution scale-unlinked (MU) planning method is similar but without any transfert of information among planning units layers (PUL). (C) The single-resolution scale-unlinked (SU) method uses only one low-resolution planning unit layer.

For the purpose of this study, the outputs of four different methods of addressing scale in spatial planning were compared (Fig 6). The comparisons were made in terms of total area and spatial distribution patterns of selected areas required to meet conservation targets (in Type A MPAs). Method 1 was the multi-resolution scale-linked (ML) method used in this study, where planning outputs from finer scales were inherited by broader scale. Method 2 was the same as Method 1 except that planning outputs from finer scales were not inherited by broader scale PUs (i.e. the multi-resolution scale-unlinked method, MU). Method 3 used only one PUL (the low-resolution 10×10 km PUL) and as a result, no finer scale planning outputs could be passed to broader scales (i.e. the single resolution scale-unlinked method, SU). Method 3 is the most commonly used methodology in spatial conservation planning. Finally, priority conservation areas identified using Method 1 (i.e. the ML) were compared with a large-scale expert-driven MPA proposal (Method 4) developed by experts from the Oceanographic Research Institute (ORI), independently from the SeaPlan process (Fig 5). The implementation of the decisions drawn from the multi-resolution scale-linked (ML) SeaPlan project in the study area are currently underway. This aspect is discussed in the Discussion section.

## Results

### Current target achievement

Overall, existing MPAs in the study area are biased toward coastal areas and the northern KZN Delagoa Bioregion (Fig 1). Of 390 biodiversity elements assigned a A target, only 48 (11%) had their target achieved in the current MPA network and only 83 (24%) of 338 biodiversity elements with a B target had their target achieved. Target achievement of biodiversity elements is summarized in Table 1 (per main categories) and S3 Appendix (per element). None of the 10 large-scale marine habitats have their A and B targets met. Areas supporting semi-permanent oceanographic processes (eddies, chlorophyll-a fronts and sea surface temperature fronts) located on the outer margin of the continental shelf have no protection. On the continental shelf, only three of nine rocky reef classes meet their conservation targets (both A and B). With only 15% (on average) of their area protected within Type A or B MPAs, the four sub-marine canyons targeted in SeaPlan are poorly protected. Sixteen of 27 coral reef classes achieve A targets. Worth noting is that these 16 coral reef classes have only partial protection within Types B and C zoning of the same MPA. Along the shoreline, only 21 of the 74 fine-scale habitat classes have their targets met by the current MPA network, mostly within the iSimangaliso MPA (St. Lucia and Maputuland) in the Delagoa bioregion. Only one of the six sandy shore classes (i.e. intermediate sandy shore) achieves its Type B target. Only one of the 17 targeted estuaries meets its target, namely the Kosi River estuary in the St. Lucia MPA. The 15 estuaries located in the Natal bioregion have no protection.

**Table 1:** Biodiversity elements, planning unit layers and conservation targets.

7	Fine- scale	Medium- scale	Large- scale	Number of elements with a target	Area in MPA type A (%)	Area in MPA type B (%)	Target A (%)	Target B (%)	Number of target A achieved/ unachieved	Number of target B achieved/ unachieved
**Habitats**										
***Broad-scale habitats***				***(10 habitat types)***						
Shelf (-30m - - 200m)—A3, A4, A5, A6, B1			x	*5*	0%	0%	10%	10%	0/5	0/5
Deep (< -200m)—B2, B3, B4, C1, C2			x	*5*	0%	0%	10%	10%	0/5	0/5
***Fine-scale shoreline habitats***				***(74 habitat types)***						
Foredune	x			*6*	17%	0%	0%	10%	na	1/6
Swash-zone	x			*8*	10%	2%	0%	20%	na	2/8
High-shore intertidal zone	x			*9*	13%	2%	4%	16%	0/4	1/9
Mid-shore intertidal zone	x			*16*	7%	2%	8%	13%	0/6	3/10
Low-shore intertidal zone	x			*19*	14%	12%	7%	13%	2/7	7/12
Surf-zone	x			*10*	6%	6%	2%	16%	0/2	3/10
Sandy shore	x			*6*	7%	7%	0%	20%	na	1/6
**Semi-permanent oceanographic processes**				***(3 oceanographic processes)***						
Eddies			x	*1*	0%	0%	10%	10%	0/1	0/1
Chlorophyll-a fronts			x	*1*	0%	0%	10%	10%	0/1	0/1
Sea surface temperature fronts			x	*1*	0%	0%	10%	10%	0/1	0/1
***Rocky reefs***				***(9 habitat types)***						
Nearshore (0 - -30 m)		x		*4*	14%	16%	10%	10%	2/4	2/4
Shelf (-30m - -200m)		x		*5*	4%	5%	10%	10%	1/5	1/5
***Sub-marine canyons***				***(4 canyons)***						
Canyons		x		*4*	15%	0%	50%	50%	0/2	0/2
***Coral reefs***				***(27 habitat types)***						
North complex		x		*9*	97%	3%	51%	33%	9/9	9/9
Central complex		x		*11*	0%	51%	55%	31%	0/11	4/10
South complex		x		*7*	99%	0%	50%	33%	7/7	6/6
***Estuaries***				***(17 estuaries)***						
Delagoa	x	x		*2*	50%	0%	0%	20%	na	1/2
Natal	x	x		*15*	0%	0%	0%	20%	na	0/15
**Species**										
***Fish (excluding sharks)***				***(66 species)***						
Fish Distribution Area (FDA)		x		*(45 species)*	6%	4%	22%	4%	0/37	0/9
Fish Species LIfe Cycles Envelops (SCLICE)				*(21 species)*						
Fish Adult Area (FAA)		x		*19*	3%	3%	20%	4%	0/15	0/4
Fish Spawning Area (FSPA)		x		*19*	4%	3%	22%	3%	0/17	0/4
Fish Nursery Area (FNA)		x		*11*	3%	2%	18%	7%	0/7	0/4
Fish migration Pathway to Adult Area (FPAA)		x		*1*	2%	1%	20%	0%	0/1	na
Fish migration Pathway to Spawning Area (FPSPA)		x		*10*	1%	1%	24%	0%	1/10	na
Fish migration Pathway to Nursery Area (FPNA)		x		*9*	1%	1%	20%	6%	0/7	0/3
***Fish (sharks)***				***(14 species)***						
Shark Distribution Area (SDA)		x		*10*	4%	4%	18%	6%	0/10	0/5
Shark Species LIfe Cycles Envelops (SCLICE)		x		*4*						
Shark Adult Area (SAA)		x		*4*	1%	2%	6%	21%	0/2	0/4
Shark Mating Area (SSPA)		x		*2*	9%	7%	15%	5%	0/2	0/1
Shark Nursery Area (SNA)		x		*3*	0%	0%	33%	0%	0/3	na
Shark migration Pathway to Adult Area (SPAA)		x		*3*	2%	2%	12%	15%	0/2	0/2
Shark migration Pathway to Spawning Area (SPSPA)		x		*3*	2%	2%	31%	0%	0/3	na
Shark migration Pathway to Nursery Area (SPNA)		x		*2*	2%	2%	20%	0%	0/2	na
***Turtles***				***(2 species)***						
Turtles nesting sites	x			*2*	27%	47%	20%	60%	2/2	0/2
***Cetaceans***				***(4 species)***						
Ceataceans Distribution Area (MDA)		x		*4*	3%	3%	16%	16%	0/4	0/4
Ceataceans Migration Pathway (MMP)		x		*1*	8%	8%	20%	0%	0/1	na

Categories of biodiversity elements are allocated to a planning unit layer (PUL). For each category of biodiversity elements, the table shows the number of targets achieved and the number of targets set. The average proportion of current distribution area of biodiversity elements in MPA (type A, B and C) is provided per category of biodiversity elements. Targets are expressed as a percentage of the current distribution area.

None of the 66 species of fish or 14 shark species included in this analysis achieves their conservation targets in existing MPAs. On average, 10% of the distribution areas of 45 fish species with mapped distribution areas [38] are included within Type A or B MPAs. A detailed analysis of the 21 species of fish with mapped Species Life Cycle Envelopes (SLICEs) shows that sites supporting key life stages are insufficiently protected [38]. On average, only 3% of fish nursery distribution areas are currently protected within Type A MPAs. Apart from the spatial extent of the “migration pathway to spawning area” of Petrus rupestris that is included within the iSimangaliso MPA, a very small proportion (less than 2%) of the migration pathways that spatially link different fish life cycle stages sites is currently protected. Results for the 14 species of sharks reveal similar trends with less than 8% of their distribution areas located within Type A or B MPAs. Two noticeable exceptions are Carcharhinus obscurus (26% of its distribution area falls within Type A or B MPAs) and Carcharias taurus (32% of its mating area falls within Type A or B MPAs). Turtle nesting sites are well protected with 74% of their distribution area falling within Type A or B zones of the iSimangaliso MPA. Turtle distributions at sea were not considered in this analysis. Less than 6% of cetacean distribution areas are covered by Type A or B MPAs, and only 16% of the migration pathway of the humpback whale is currently protected (within the iSimangaliso MPA).

### Index of cumulative anthropogenic pressures

**Fig 7:**
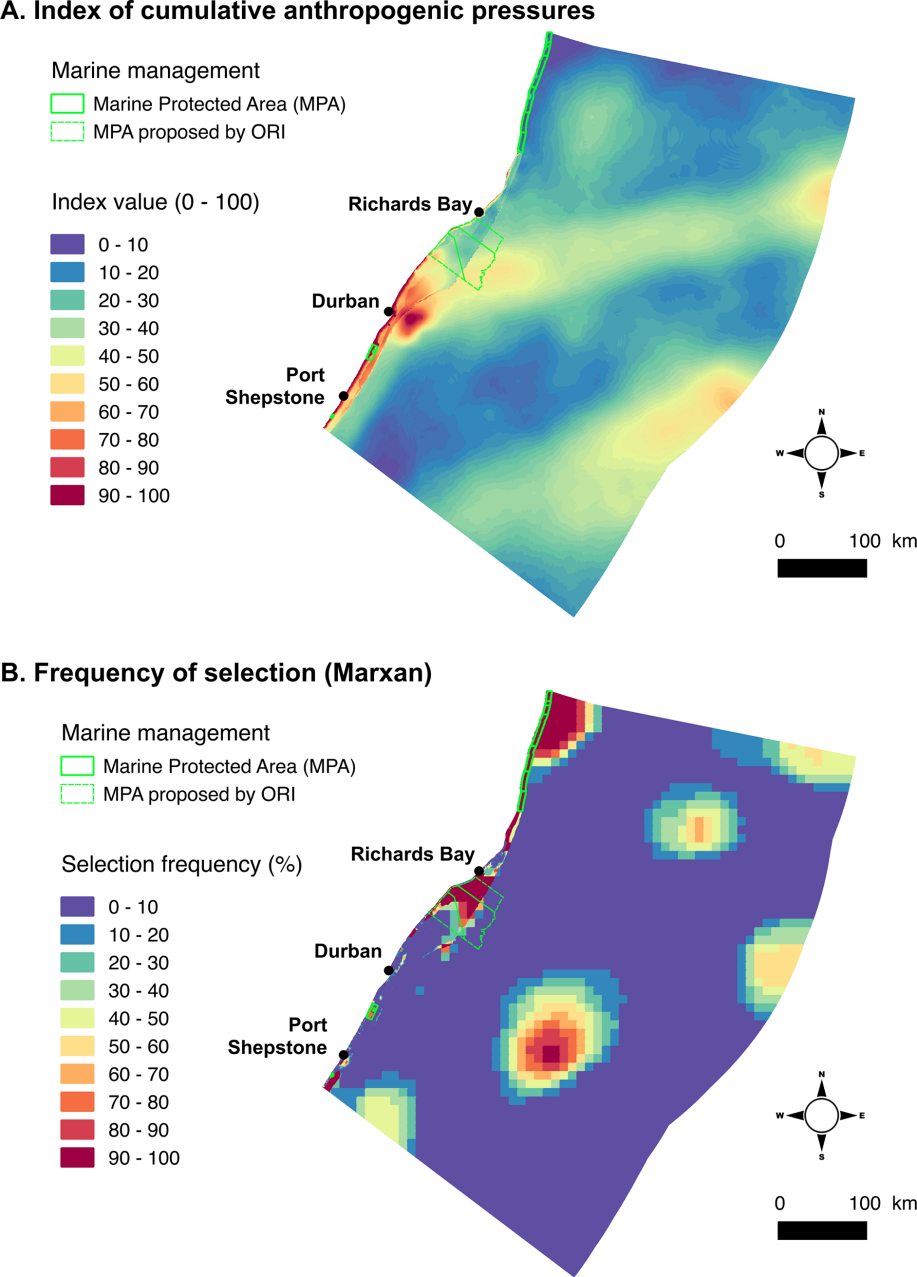
Maps of index of cumulated anthropogenic pressures and priority sites for conservation. The map of index of anthropogenic pressures indicates areas under human-induced impacts from blue (low impact) to red (high impact) (A). The map of selection frequency (maximum value) along MARXAN runs (at fine, medium and large-scale) indicates higher priority areas for conservation from blue (low priority) to red (high priority) (B).

The mean value for the index of cumulated anthropogenic pressures across the entire study area is 15 (standard deviation = 6, minimium = 0, maximum = 100,) (Fig 7A). Areas with high index values (≥40) are distributed along the shoreline close to the main coastal cities (i.e. Durban and Richards Bay), and in the vicinity of harbours and industrial pipelines, and in easily accessible coastal areas. High pressure areas are also located offshore in zones targeted by industrial fishing vessels and along major shipping routes linking Durban to ports located in East Asia. Not surprisingly, low pressures areas (close to 0) are located within Type A and B MPAs. It is noticeable that pressure scores inside Type C MPAs are higher than within adjacent areas located outside the MPA [27], due to the presence of attractive recreational infrastructures (e.g. parking access and boat launching sites for instance).

### Proposed priority areas for conservation

This section presents the best solution for a spatial network of protected areas identified using the multi-resolution scale-linked (ML) planning method to achieve the full set of conservation targets (Fig 7B, Table 2). Currently only 0.1% of the planning domain is made up of Type A MPA zones. A total of a further 11.3% (26,341km^2^) of the planning domain is selected as priority areas to achieve Type A targets. Only 16.8% of the shallowest depth zone 1 is selected; whereas greater than 40% of depth zones 2 and 3 were selected (43.5 and 48.6%, respectively). Only 9.8% of depth zone 4 is selected, reflecting the lower targets for habitats in the deeper zone.

**Table 2:** Area-based comparison of protected areas networks designed using three alternative multi-resolution conservation planning procedures.

Depth zone (DZ)	Total area (km^2^)	Area conserved (km^2^) (% of zone area)	Spatial planning procedure	Area selected (km^2^) (% of DZ area)	Difference to area selected in ML (km^2^) (% of benchmark area)
**DZ 1**	140	17 (12.2%)	SU	54 (38.6%)	+31 (+125%)
			MU	24 (16.8%)	0 (0%)
			ML	24 (16.8%)	(benchmark)
**DZ 2**	1,646	77 (4.7%)	SU	1,417 (86.1%)	+701 (+97.9%)
			MU	706 (42.9%)	-10 (-1.4%)
			ML	716 (43.5%)	(benchmark)
**DZ 3**	7,115	163 (2.3%)	SU	4,105 (57.7%)	+649 (+18.8%)
			MU	1.840 (25.9%)	-1,616 (-46.8%)
			ML	3,456 (48.6%)	(benchmark)
**DZ 4**	224,985	12 (<0.1%)	SU	22,368 (9.9%)	+223 (+1%)
			MU	22,368 (9.9%)	+223 (+1%)
			ML	22,145 (9.8%)	(benchmark)
**DZ 1,2,3,4**	233,887	269 (<0.1%)	SU	27,945 (11.9%)	+1,604 (+6.1%)
			MU	24,938 (10.7%)	-1,403 (-5.3%)
			ML	26,341 (11.3%)	(benchmark)

Area of planning domain zones (in km^2^ and in percentage) selected using three alternative multi-scale spatial planning methods: single-resolution scale-unlinked (SU), multi-resolution scale-unlinked (MU) and multi-resolution scale-linked (ML). The ML output is used as benchmark for comparison. DZ 1, 2, 3 and 4 are depth zones from the inshore to the offshore with the following limits: Vegetation line, -2m, -30m, -200m and outer boundary of the Exclusive Economic Zone. Outputs were obtained using the MARXAN best solution for A targets.

Priority areas were identified incrementally for the fine-, medium- and large-scale PUL’s. The fine-scale PUL covers an area of 1,786 km^2^ and includes depth zones 1 (140 km^2^) and 2 (1,646 km^2^) (Fig 3), and consists entirely of high resolution PUs. A proportion of 12% of Zone 1 has Type A MPA protection. In the conservation plan, an additional 17% is selected to achieve Type A conservation targets for estuaries and fine-scale shoreline habitats. These additional priority areas are selected on sections of coastline with a high diversity of shoreline habitat types, and in the vicinity of an estuary (Fig 5, S1 Appendix) and a low impact score (S2 Appendix). Some shoreline habitat types are rare and this causes additional isolated sites to be selected along the shoreline. A small proportion (5%) of zone 2 has Type A MPA protection and 65 km^2^ (4%) is selected as part of the ‘best solution’ in the high-resolution PUL, including 62 km^2^ of existing Type B protection located within the iSimangaliso MPA (the additional 3 km^2^ required is to achieve conservation targets for the marine extent of estuaries).

The medium-scale PUL covers an area of 8,762 km^2^ and includes depth zones 2 and 3 (Fig 3). This PUL overlaps with the high-resolution PUL in zone 2. In this zone 2, 8% of the area is pre-selected for the conservation plan run, because a number of planning units overlap by more than 50% with the “best-solution” identified by the conservation plan run at the finer scale. In addition to this 8%, another 35% is selected to achieve Type A conservation targets. In zone 3, 163 km^2^ (2% of this zone) is covered by existing Type A MPA and an additional 18% is selected to achieve Type A conservation targets. At this stage of the site selection procedure, conservation targets are achieved for the following medium-scale elements: rocky reefs, coral reefs, submarine canyons, large estuaries, fish and cetaceans species (Fig 5, S1 Appendix). A visual analysis of the spatial patterns of priority areas shows that the northern part of the Natal Bight (North of Durban) is a focal area to achieve conservation objectives for elements included in the medium-scale PUL (Figs 4, 5 and 6B). This area combines a high diversity of fish, coral reef habitats and the influence of the Mzimkhulu and uThukelaRivers. In the south, close to Port-Shepstone, the spatial clusters of priority areas linking the shoreline to the -200 m depth line offshore are explained by the proximity of targeted estuaries, submarine canyons and higher fish species richness (Figs 4, 5 and 6B, S1 Appendix).

The large-scale PUL covers an area of 224,985 km^2^, from the -30 m depth line up to the offshore limit of the EEZ (zones 3 and 4 in Fig 3). Currently, there are no MPAs in zone 4. The large-scale PUL overlaps with the medium-scale PUL in zone 3 and sites selected from the medium-scale PUL cover an area of 1,087 km^2^ (15% of this zone). Selected additional sites for conservation cover an area of 25,372 km^2^, including 3,229 km^2^ in zone 3 (46% of this zone) and 22,143 km^2^ in zone 4 (10% of this zone). Priority areas in zone 4 are associated with semi-permanent eddies (Figs 5 and 6B, S1 Appendix). Priority areas in zone 3 are spatially aggregated on the northern extent of the Natal Bight, expanding southward to integrate areas supporting semi-permanent sea surface temperature fronts bordering the landward side of the Aghulhas current (Figs 5 and 6B, S1 Appendix).

### Comparison of multi-scale planning methods

The SU, MU and ML methods select 27,945 km^2^, 24,938 km^2^ and 26,341 km^2^ for conservation respectively (Table 2, Fig 6). Using the ML method as a benchmark, the SU method selects more area (1,604 km^2^, +6%) and the MU method less area (-1,403 km^2^, -5%) to achieve the same conservation targets. As would be expected, the SU method selects large areas in coastal zones owing to the large size of planning units (10×10 km) where the MU and ML methods have smaller planning units (0.2×0.2 km and 1×1km) that better match fine and medium scale distribution data of high and medium resolution. For instance, along the coastline, the SU method selects 39% of zone 1, versus only 17% using the MU and ML method. With the ML method, the mandatory selection of finer-scale PUs adds these PUs to those needed to achieve conservation targets at the broader scales in areas that may not match spatially. While requiring slightly more area to achieve targets compared to the MU plan, the ML plan ensures a spatial continuum of priority areas linking the inshore to the offshore domain, which was an initial objective of SeaPlan (Fig 7B). This continuity is not achieved in the MU plan with a more spatially scattered ‘best solution’. We interpret the additional area selected in the ML as the spatial cost of linking inshore and offshore conservation priorities.

Priority areas for conservation identified using the ML method were compared with a proposed MPA delineated by marine conservation scientists at ORI, who used a more intuitive expert knowledge-driven, and not a systematic conservation planning approach. Fig 5 and Fig 7B show good spatial overlap between the initial ORI proposal and the priority areas (best solution and selection frequency) identified by SeaPlan on the uThukela banks using the ML method. However, the SeaPlan output was generally more spatially efficient representing a subset of a greater area with core priorities within the focal area highlighted.

## Discussion

### Why multi-scale spatial planning?

The need to develop multi-scale approaches to marine spatial planning has been advocated to account for mismatches between the nested and multiple scales of biodiversity pattern and process variation through space, the disparate scales over which different anthropogenic impacts operate, and the scales (local to global) at which interventions are proposed. In the inshore, particularly the shoreline, scales of change in biological patterns are generally much finer than offshore, and stakeholders are strongly invested in local issues and interested in patterns of the biodiversity that are clearly visible to them. Accounting for, and generating action to respond to, the many diverse local conservation issues within the development of broader scale plan, without obfuscating them by absorbing them in lower resolution information packages, is a crucial but complex task. To address this challenge, this study developed and tested a method for identifying priority areas for conservation simultaneously and systematically at fine, medium and broad scales, across the whole planning domain from the shoreline to the offshore limit of the EEZ (covering coastal and offshore environments), while taking into account the nature and scale of anthropogenic drivers of ecosystem change. This multi-scale multi-resolution spatial planning method was developed and tested in the preparation of a marine conservation plan for the eastern seaboard of South Africa (the SeaPlan project), but provides an approach of general relevance.

Like many countries, previous marine conservation assessments and plans in South Africa have been undertaken independently at different scales (e.g. national, provincial, local), using data of different resolutions aggregated to a single resolution, and addressing various components of the marine environment (e.g. bioregions, inshore/offshore, benthic/pelagic) and human use patterns (e.g. fishing pressure) seperately. A national marine assessment providing a very broad framework for biodiversity conservation planning exists, but it is limited by the scarcity of consistent fine-scale data that cover the whole planning region [26, 27]. As is the trend world-wide, the existing MPAs within the study area are strongly biased towards the coast with offshore protection being virtually non-existent. The extent and distribution of the coastal MPAs is also biased, with high protection in the northern Delagoa bioregion and little protection in the Natal bioregion. Unsurprisingly, the highest levels of anthropogenic pressures are experienced along the shoreline, close to the main cities (Durban and Richards Bay) (Fig 7A). This has two implications: first, many options for marine conservation are still available in the offshore domain (but these options are starting to foreclose owing to escalating offshore activities) and, second, inshore conservation actions are severely spatially constrained. Priority areas for conservation shown on Fig 7B can serve as a basis for future MPA expansion.

In comparison with the offshore, the implementation of shoreline and inshore marine protected areas require finer scale identification of spatial priorities (to improve efficiency and limit conflict with users in a crowded space), more rapid action and opportunistic timing (to get ahead of looming fore-closure on options), and the ability to demonstrate tangible linkages between condition of biodiversity and benefits of protection (to garner local stakeholder support). The multi-scale nature of our method, which explicitly identifies and assigns spatial conservation priorities along the shoreline and the inshore first, embeds these priorities as key drivers of the selection process for offshore spatial conservation priorities. This ensures that the priorities in the area of the planning domain that has least options for protection and most pressure are “hard-wired” into the plan up front, while spatial continuity is achieved by adjacent selection of priorities in the areas where more options exist for meeting conservation targets, and where there is greater scope for adaptation.

### Improving the spatial efficiency of marine zoning

Efficiency in space allocation for different uses of the ocean is an important consideration in an ocean that is becoming crowded with competing activities [1, 2]. Efficient spatial planning has the added advantage of facilitative achievement of efficiencies in deployment of management capacity. We found that our new ML spatial planning approach was more efficient compared to a conventional SU method, and in our case study 6% less area was selected to achieve the same conservation targets. This result is owing to the selection of only the smaller (high-resolution) priority planning units in the ML method, compared to selection of the larger (low resolution) planning units within which small-scale elements are absorbed in a SU plan. Compared to a scale-unlinked version (MU) of the multi-resolution approach, the ML plan required more area (5%) to meet conservation targets. This apparent reduced spatial inefficiency is however mitigated by the gains in terms of ecological connectivity achieved between identified priority conservation areas that occur across different spatial scales.

To promote efficiencies, many spatial conservation planning exercises entrench existing MPAs as mandatory for selection, thereby reducing the selection of new areas to achieve targets. In the SeaPlan case-study, the ML approach reinforced this effect, because existing MPAs in the planning area are all coastal. By prioritising expansion of the existing coastal MPAs over the creation of new and isolated MPAs, the positions of the coastal MPAs strongly influenced selection of offshore priority areas to achieve targets. The resultant ML spatial plan meets most conservation targets by expanding existing coastal MPAs into the offshore environment (Fig 6).

In the study area, some conservation targets can be addressed simply by rezoning appropriate areas of current MPAs (for instance the coral reefs in the Delagoa region). Additional coastal protection is required in the south of the Natal bioregion, again best achieved adjacent to the existing MPAs. In the offshore, the plan identified unique areas that are distant from existing MPAs, and merit conservation management. In the southern offshore, the area of interest is defined by the Protea Banks reef system and associated deep canyons on the continental shelf edge, where the dominant southward flowing warm Agulhas current moves close inshore. Further north, still within the Natal bioregion, an additional large MPA should be implemented to conserve a spatial ecological continuum from the uThukela River up to the offshore limit of the KZN Bight, thus linking up with the semi-permanent sea surface and chlorophyll-a fronts associated with the Agulhas current (Fig 5). The resulting MPA network should contribute to the persistence of ecological processes across ecological realms, along the terrestrial-marine-freshwater continuum [73].

### Strenghtening political buy-in and social acceptance related to marine conservation

Adopting a ML method reduced political risk (loss of political support) owing to regular interactions with decision makers and stakeholders across spatial scales. The multi-scale approach ensured a strategic and stable positioning of the project on the political agenda at national, provincial and local scale over years, throughout a constantly changing social and political landscape. This was also made possible through a deep rooting of the SeaPlan project within a single conservation planning agency (EKZNW), that had the required capacity at a provincial level and was committed to contribution to national objectives.

Once the results of SeaPlan emerged, EKZNW prepared a MPA Expansion Plan based on the results, and motivated to the national Department of Environmental Affairs to start the process of proclaiming new and expanded MPAs. The national Department supported an initial process of stakeholder engagement to develop proposals for two new MPAs, over the uThukela Banks and the Protea Banks. This process was then absorbed in 2014 into a national process initiated by the Office of the Premier to fast-track the unlocking of the Ocean Economy in South Africa (i.e. Operation Phakisa). Phakisa was aimed at stimulating commercial industries, but also provided an important opportunity to articulate the potential negative impacts and risks of rapidly emerging industries such as mining and aquaculture, and to consider mitigation measures. Phakisa gained political and public support to initiate the establishment of an expanded network of MPAs, to increase offshore protection within South African waters from 0.4% to 5% within 3 years.

Of the twenty-one areas included in the Phakiza proposed national MPA network, which was refined from existing products in very short period (six weeks) in 2014, four had been identified by the SeaPlan project. The rapid devlopment of this proposed MPA network design was possible owing to the high state of technical readiness amongst the team of conservation planners working collaboratively across scales, as well as to the availability of existing spatial products developed collectively by the country’s marine conservation planners over the preceding decade [60, 63, 64]. It was also facilitated by an established dialogue between national and provincial government agencies, because the SeaPlan priority areas had already been submitted to and supported by national government and embedded in the national planning processes.

The concurrence and synergies between the coarse large-scale national MPA expansion priorities, and those identified by SeaPlan at a finer scale, meant that there was confidence in including overlapping areas such as Protea Banks and uThukela in the Phakisa MPA network. In addition, the expansion of the Aliwal Shoal and iSimangaliso MPAs were also validated and included in the Phakisa MPA network. These successes demonstrated the value of working at different scales, both with respect to technical analyses and decision-making spheres.

Although this study doesn’t emphasize the participation of stakeholders, they were strongly involved in the SeaPlan planning process. This participation is vital for the development and implementation of conservation plans and it guarantees its legitimacy [4, 8, 26]. Among the benefits of the multi-scale approach, is that it provides for understanding by stakeholders of conflicts between and synergies amongst their spheres of interest and influence, and those of other stakeholders who they previously regarded as unrelated or unapproachable. We have found that stakeholders are more strongly invested in knowledge at a scale relevant to their specific activity, for example shore users showed a strong interest in localised intertidal areas that were clearly visible and known to them (at a scale of 100 m-10 km), whereas boat-based fishers, while interested in the shoreline from the perspective of safe launch sites, had a larger area of interest (10–100 km) and were less interested in detailed elements at the scale of hundreds of metres. Similarly, further offshore trawlers (who operate at scales of >10 kms) and seafloor mining (that operates at scale of >100 km) had overlapping areas of interest for their activities but were not invested in the fine scale features of the plan. By analysing and displaying the data and plan at multiple scales, such users were able to articulate and engage with one another about the relative impacts of each other’s activities on one another as well as proposed management actions. This facilitated dialogue about overlaps, conflicts and common interest, and created stakeholder interest and alliances in broader scale planning issues, thus adressing constraints identified by [73]. This interplay among a wide array of sea users operating at different scales strengthened the SeaPlan spatial process.

### Future uses and developments of the method

Conducting spatial planning across multiple scales involves additional costs to achieve expected benefits, compared with single resolution spatial planning, specifically with respect to the costs of time and expertise for data collection, analysis and stakeholder engagement [74]. The ML method used in this study involved more stakeholders (and at different levels) and required more frequent and complex interactions with them. While we are currently unable to translate these costs into monetary values, we believe that any additional costs need to be seen in the context of the long-term benefits gained from the ecosystem services supported by cross realm planning [75].

The implementation of the ML method, compared with other methods (SU and MU), resulted in a better overall spatial fit of the proposed MPA network to biodiversity targets and constraints across coastal and offshore marine realms [75]. The benefits of this method are more easily assessed in terms of planning cost avoidance: SeaPlan tackled fine, medium and broad scales within a single spatial planning process, thus avoiding the cost of three separate spatial plans. In addition, the ML method made the best use of all available data and no time was wasted in collecting data that were not used in the planning process. Comparison of the ML plan with the expert-driven plan showed good congruence, although the ML-derived plan was more conservative in the size of the area selected for protection. Whereas, the expert-method was applied only in a restricted area generally considered to be of conservation significance, the ML method had the advantage of being systematically replicable over the entire planning domain, and its selection procedure was explicit.

The ML method contributes to building technical solutions to scale mismatch problems that impede effective implementation of spatial plans [4, 5, 6]. For instance, the difficulty of mixing Integrated Coastal Zone Management and broader Marine Spatial Planning [76] can be conceptualized as a case of scale mismatche that could be addressed using the ML method. The ML method is relatively simple and can act as a technical bridge to link distinct conceptual, technical and social processes operating at different scales [4]. In a rapidly changing world, this marine spatial planning approach addresses the urgent need for integrating coastal and offshore conservation strategies [1, 2, 3]. Coordination across scale does not increase the area selected to achieve targets by much, but it does support better choices in spatial allocation of marine management actions.

Future developments of the ML method should focus on coupling the social and technical dimensions of the multi-scale planning process [77]. Another direction to explore is the development of routines and data formats within current GIS software and spatial optmization tools to ease multi-resolution data management and analysis. The time invested in the organisation, formatting and analysis of multi-scale multi-resolution data could then be invested in more effective tasks. We also suggest that post-hoc assessments be done of marine spatial plans, across scales, to ensure that they are self-consistent.

## Conclusions

Multi-scale multi-resolution spatial planning is challenging. The methodology developed in this study allows the identification of priority areas for conservation at fine, medium and large scales within a single multi-scale multi-resolution spatial planning framework. The method enables incorporation of diverse biodiversity elements, ecological processes and human use patterns, that occur or operate across different scales and vary across their distribution at different rates, while at the same time maintaining the data resolution integrity for these elements. This approach reflects the multi-scale nature of coupled social and ecological systems.

Our multi-resolution scale-linked (ML) planning method delivers a more efficient spatial solution for achieving conservation targets within marine protected areas, than a single resolution scale-unlinked plan. Whereas the ML method requires slightly more area, when compared with a multi-resolution scale-unlinked method, it promotes the ecological connectivity of the future MPA network across spatial scales. Although the monetary costs of developing and implementing a ML spatial plan compared to scale-unlinked methods were not quantified, the ecological, social and political benefits (and cost avoidance) of the ML method are likely to outweigh any additional costs that may be incurred for skills, data collection and social interactions.

This study provides a useful, repeatable and transferable approach that is applicable to multi-scale spatial planning in general. It provides a methodological development that has the potential to transform conservation planning practices and policies in many marine regions worldwide. This very simple idea was technically difficult to implement, and time consuming, but has delivered a plan that applies to the real world (by linking social and ecological systems more tightly) and thus has increased potential to achieve political support and implementation across different management spheres. Future technical developments of the method should focus on easing the data management, and advancing the modelling and analytical processes, exploring sensitivity to additional extraneous factors, and cost-benefit analyses. Further development and refinement of methods to integrate social and ecological dimensions in multi-scale marine spatial planning is important, particularly if we wish to address real-world, complex planning problems. These efforts should involve interdisciplinary teams, so that the development of sector-specific outputs is avoided, and integrated multi-sector spatial plans are enabled and promoted.

Our study shows the positive impact of a multi-scale and self-consistent approach to marine spatial planning in terms of political and social buy-in at local ad national level. The overall approach proposed in this study is a meaningful contribution to marine spatial planning in general, i.e., not only in terms of methodology or from a conservation point of view. We hope this study will contribute to help societies developing sustainable futures througout marine spatial plans taking into account the increasing interactions among human activities and biophysical phenomenon across multiple spatial scales, from earth-scale down to local-scale.

## Supporting information

S1 Appendix Data and methods for mapping biodiversity elements in the SeaPlan planning domain. (PDF)

S2 Appendix Data and methods for mapping human activities and the index of anthropogenic pressures in the SeaPlan planning domain. (PDF)

S3 Appendix List of biodiversity elements and conservation targets. (PDF)
