# Supplementary material for: Multi-scale multi-level marine spatial planning: A novel methodological approach applied in South Africa
Source: PLoS One. 2018 Jul 3;13(7):e0192582. doi: 10.1371/journal.pone.0192582 (PMC6029778; doi:10.1371/journal.pone.0192582)
Supplement: S1 Appendix — (PDF) [file pone.0192582.s001.pdf]

S1 Appendix. Data and methods for mapping biodiversity elements in the SeaPlan project
Adapted from [1].

1. Broad-scale elements

1.1. Bathymetry

Bathymetry data were obtained from Paul Young at the UKZN Marine Geosciences. [2] compiled an extensive dataset for KZN producing a bathymetric grid and contour lines. A total of 32 datasets were acquired to develop this map using a range of techniques and instruments used between 1911 and 2006. Twenty nine of these were near-shore datasets with data densities varying from 6 to 57 406 points per km^2, 15 were acquired from the Council for GeoScience, 9 from the South African Navy and 5 from the African Coelacanth Ecosystem Programme (ACEP). Two of the remaining 3 deep-water datasets were grids acquired digitally for this work, while the third was a digitised contour dataset. The 2003 General Bathymetric Chart of the Oceans (GEBCO) grid is based on digitised point and contour data with a point every 1 852 m [3], while the 1997 Smith and Sandwell grid, is based on predicted satellite altimetry data with a point every 3 704 m [4]. The third deep-water dataset was digitised from a northern Natal Valley bathymetric contour map developed in 1978 and has data densities varying from 0.02 to 1 points per km^2 [5]. Data were processed by Marine Geosciences by interpolating a point surface to 500 m resolution. A 100 m resolution surface was then interpolated from this data set, and clipped to the exclusive economic zone (EEZ) extent (i.e. from the KZN high-water mark, out to 200 nautical miles, bound to the provincial waters) (Fig 1).

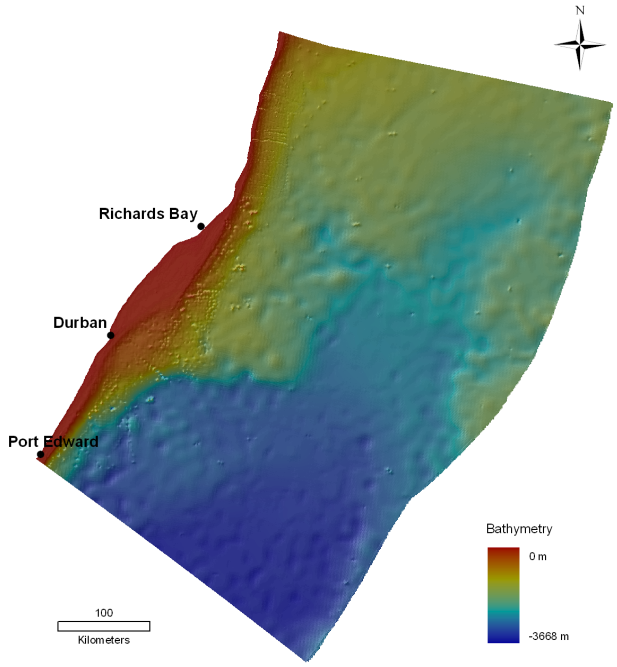

Fig 1. Bathymetry of the planning domain (overlaid over relief).

1.2. Marine habitats

Twelve broad-scale marine habitat units (average surface of 2297 km2 per habitat type) were mapped at 1 km resolution to serve as spatial surrogates for marine biodiversity distribution at regional scale. The mapping method forms part of a study by Livingstone et al. (unpublished). The initial steps of the method consisted in selecting the most appropriate variables to create a biologically meaningful classification of the ocean water masses. The choice of the variables and parameters, made by a panel of scientists consulted in Seaplan, is assumed to reflect the drivers of the distribution of biodiversity patterns and processes. The variables and parameters used were: SST mean, SST max, SST coefficient of variation (CV), Chlorophyl-a (Chl-a) mean, Chl-a CV, Turbidity mean, Turbidity CV bathymetry and slope. Data used for clustering were extracted from pathfinder AVHRR sea surface temperature data (SST), SeaWiFFs chlorophyll-a data and K490 AquaModis (turbidity) data. Bathymetry data at 100m resolution were extracted from [1]. Input datasets are described in Table 1.

Table 1. Summary of data used to map broad-scale habitats based on a k-mean clustering analysis

Variable/Feature	Parameter/Treshold	Method
Bathymetry	[value], [slope]	Bathymetry data at 100m resolution extracted from [2].
Sea Surface Temperature (SST)	[mean], [maximum], [coefficient of variation]	Data extracted from NOAA/AVHRR SST data, 1 km resolution, Jan 2001 to Dec 2004. Data processed and provided by the Oceanography Department of the University of Cape Town (C. Whittle). Best cloud free image per month for a total of 46 images.
Chlorophyll-a	[mean], [coefficient of variation]	Data extracted from SeaWiffs chlorophyll (Chl-a) data, 1 km resolution, Jan 2001 to Dec 2004. Data processed and provided by the Oceanography Department of the University of Cape Town (C. Whittle). Best cloud free image per month for a total of 51 images.
Turbidity	[mean], [coefficient of variation]	Data extracted from AquaModis diffuse-attenuation coefficient for PAR (m-1), 4 km resolution resampled to 1 km, Jul 2001 to Dec 2004. Data downloaded from the NASA oceancolor website (http://oceancolor.gsfc.nasa.gov/cgi/level3.pl). Monthly composite data for a total of 30 images.
Sediment type	[Type]	Mapped from [6] over the KZN continental shelf.
Phosphate	[Concentration]	Mapped from [6] over the KZN continental shelf.
Organic carbon	[Concentration]	Mapped from [6] over the KZN continental shelf.
Seabed oxygen	[Concentration]	Data provided by the Department of Oceanography of the University of Cape Town (F. Duncan), South African Data Centre for Oceanography (SADCO), Marine and Coastal Management (MCM) and Bayworld Centre for Research and Education (BCRE).
Seabed temperature	[Value]	Data provided by the Department of Oceanography of the University of Cape Town (F. Duncan), South African Data Centre for Oceanography (SADCO), Marine and Coastal Management (MCM) and Bayworld Centre for Research and Education (BCRE).

A K-mean cluster analyses was performed in the statistical program R on the above-mentioned variables. Three main clusters ("level-1 clusters") were identified. Cluster A is the continental shelf (from 0 to approximatly -200 m) with high productivity water. Cluster B depicts the spatial extent of the Agulhas current with low productivity warm water. Cluster C is a deep water mass caracterised by low productivity and higher surface variability. Further K-mean analyses were performed within each of the clusters A, B and C in order to reflect the subtle internal patterns of temperature and productivity. Clusters B and C were respectively divided into four (B1, B2, B3, B4) and two sub-clusters (C1, C2). The cluster A was sub-divided into 4 sub-clusters (A3, A4, A5 and A6) based on a K-mean analysis applied to the following variables: bottom sediments (mud, silt, clay, sand and gravel) mapped from [2] and seabed oxygen and temperature (Fiona Duncan, UCT, pers. comm.). Two additional clusters (A1, A2) were delineated based on bathymetry (0 - 30m) in the Delagoa and Natal bioregion. Note that A1 and A2 were not allocated a conservation target in SeaPlan. The final map is a seamless regional coverage made of twelve broad-scale habitat units (Figure 2).

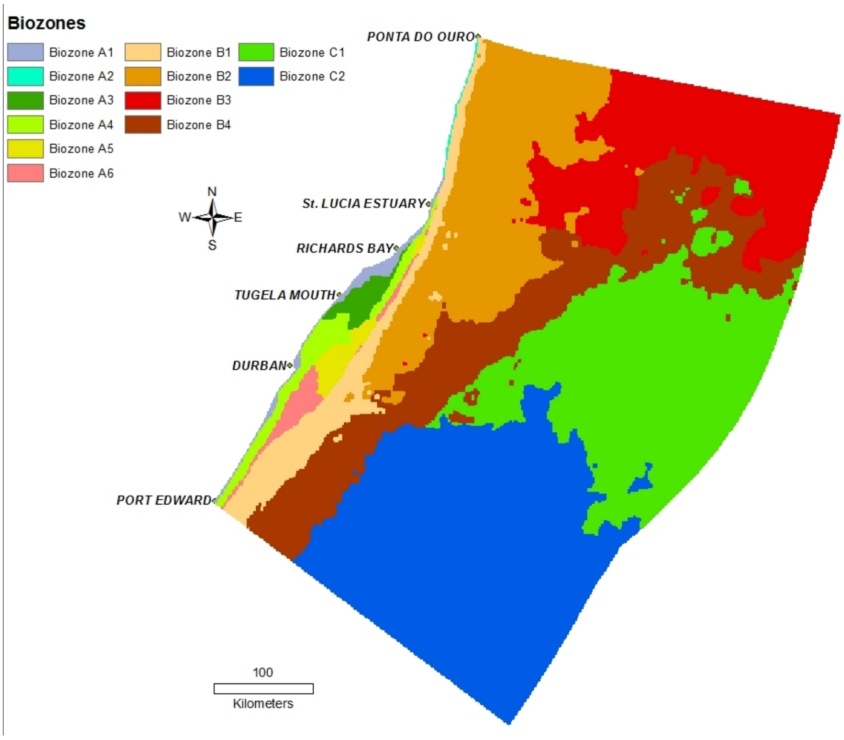

Fig 2. Spatial distribution of the 12 broad-scale marine habitat types (i.e. clusters) in the Kwazulu-Natal province exclusive economic zone.

1.3. Oceanographic processes (fronts, eddies, currents)

Medium-scale oceanographic elements such as mesoscale eddies, upwellings and fronts have an important impact on the exchange of biophysical material and energy in the ocean and are often associated with higher primary productivity. In the Mozambique Channel, it was demonstrated that top predators, such as seabirds and tuna, tend to concentrate in areas with slightly enhanced productivity such as a zone of strong eddies [7]. Available techniques for the extraction of oceanographic elements range from photo-interpretation, to more complex automatic extraction techniques such as object-oriented extraction methods [8]. In this study, we used a front detection algorithm on sea-surface temperature and chlorophyll-a concentration data to map fronts, and altimetry data to map eddies over a time series [9] (Table 2, Fig 3). Areas supporting semi-permanent mesoscale fronts were mapped from SST and Chlorophyll-a 2002-2007 MODIS data at 4 km resolution. Areas supporting semi-permanent eddies were mapped using Monthly 2001-07 Mean Sea Level Anomalies data from the spatially nested oceanographic current model HYCOM [10] at 10 km spatial resolution. Cyclonic eddies are associated with negative sea level anomalies (SLA) whereas anticyclonic eddies exhibit positive SLA values. We retained an amplitude threshold of at least + or - 10 cm on altimetry data to extract cyclonic and anticyclonic eddies in the EEZ of the Province.

Table 2. Summary of data used to map medium-scale oceanographic processes (fronts, eddies, currents)

Variable/Feature	Parameter/Treshold	Methods
Semi-permanent Sea Surface Temperature (SST) fronts and Chlorophyll-a fronts	[Frequency of fronts > 50]	SST fronts and Chlorophyll-*a* fronts frequency over the 2002-2007 period were computed from 8 days MODIS data at 4 km resolution. Data downloaded from the NASA oceancolor website (ftp://oceans.gsfc.nasa.gov). Fronts were extracted using the Cayula-and Cornillon algorithm [9]. This algorithm is implemented in the Arctool Box MGET [11]. Zones with a frequency > 50 % were extracted.
Semi-permanent eddies	[Frequency of fronts > 50]	Anticyclonic and cyclonic eddies elements were extracted by applying a +-10 cm threshold over HYCOM Monthly 2001-07 Mean Sea Level Anomalies data (computed with respect to a 2001-07 mean), 10 km resolution. The frequency of eddies (both cyclonic and anticyclonic) was then calculated over the entire time series. Zones with a frequency > 50 % were extracted. Data provided by the Department of Oceanography at the University of Cape Town (B. Backeberg).
Geostropic currents	[Mean], [Eigen vectors/Principal Component Analysis]	HYCOM currents (U and V components) extracted from monthly 2001-07 Mean Sea Level data, 10 km resolution. Data provided by the Department of Oceanography at the University of Cape Town (B. Backeberg). Data used as background material.

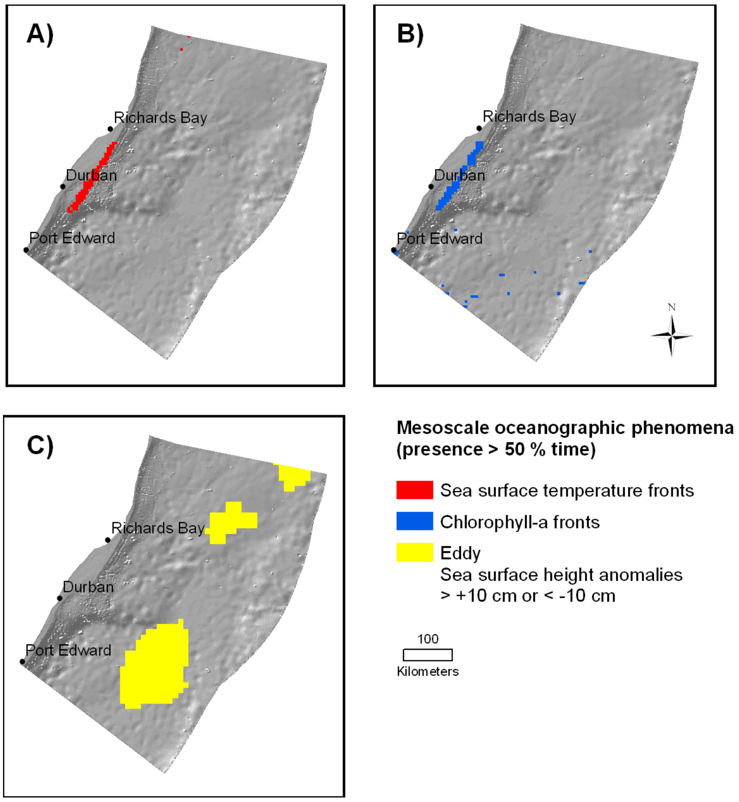

Fig 3. Spatial distribution of semi-permanent A) SST fronts, B) chlorophyll-a fronts and C) eddies (cyclonic and anticyclonic) in the Kwazulu-Natal province exclusive economic zone.

1.4. Cetacean species distribution (whales, dolphins)

Cetaceans species to be included in the conservaton plan were selected based on the following criterion: distribution range, duration of presence in KZN waters, and vulnerability of the population in the province. Four cetacean species distribution were mapped for this analysis: the Bottle nose dolphin, the humpback dolphin, the sperm whale and the humpback whale. Although several of the cetaceans like humpback whales and humpback-dolphins have been observed in open ocean waters, only their most critical inshore habitats were mapped for this project. Sperm whales are the only species that are confined to the offshore and the area from the 1000 m depth contour out to the offshore boundary of the EEZ was mapped as their habitat. Humpback whales have nearshore migration routes between their Antarctic feeding grounds and tropical calving grounds. This route was captured in a GIS layer, using the description of [9] who describes the route as predominantly between 1 - 3.5 km offshore. Dr's Peter Best and Ken Findlay were contacted to gain their insight into species selection, information on important reference works and distributions of the species. Further information was obtained through email correspondence with Ken Findlay whose PhD thesis [12] formed the primary source of information on species distributions.

2. Medium-scale elements

2.1. Geomorphological elements (canyons, rock reefs, coral reefs)

Fifty-one deep-sea canyon elements (below -200 m) were mapped using data derived from a 100 meters resolution multibeam bathymetric survey of the northern KZN continental shelf undertaken by Marine GeoSolutions (Pty) Ltd in conjunction with the National Research Foundation. This survey was used to map known submarine canyons between Leven Point and Island Rock and to provide bathymetric maps and three-dimensional models of the canyons [13] Marine Geoscience at the University of KZN provided the detailed bathymetric maps of canyons found elsewhere along the KZN continental shelf break.

Rocky reef presence was mapped down to -200 m depth (Fig 4). A shallow reef habitat survey was conducted during which positions of reefs were recorded and incorporated into the database and a coastal flight also provided GPS positions of shallow and visible reefs. This information was incorporated with rock reef location data sets obtained from the South African Navy, the Oceanographic Research Institute, and Marine Geoscience. Other distribution data on reef locations were obtained from a literature review and by conducting interviews with EKZNW management staff, students and scientists, recreational or commercial divers, line-fishers and spear fishers. Data were collated into a final raster layer (1 kilometer resolution) of rock reefs presence on the continental shelf

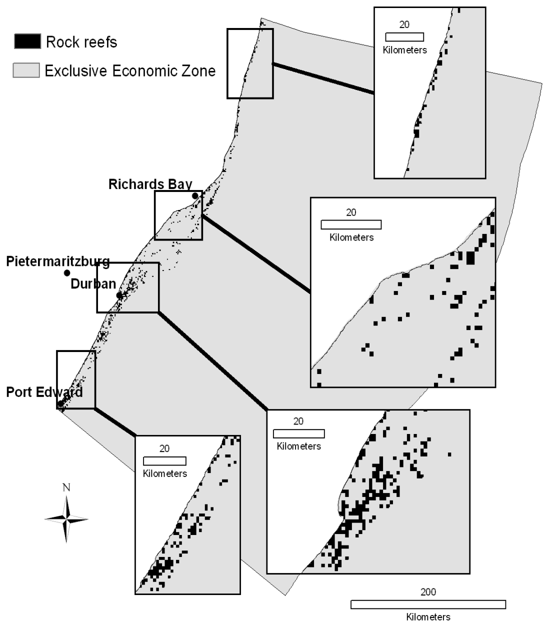

Fig 4. Spatial distribution of rocky reefs in KwaZulu-Natal. Rocky reefs were mapped for the SeaPLAN project, only between 0 and -200 m depth.

Coral distribution data (100 meters resolution grid) were provided by the Oceanographic Research Institute [14] . Twenty-seven coral reefs types belonging to three sub-regional coral reefs complexes (Northern, Central, Southern) were differentiated (Fig 5).

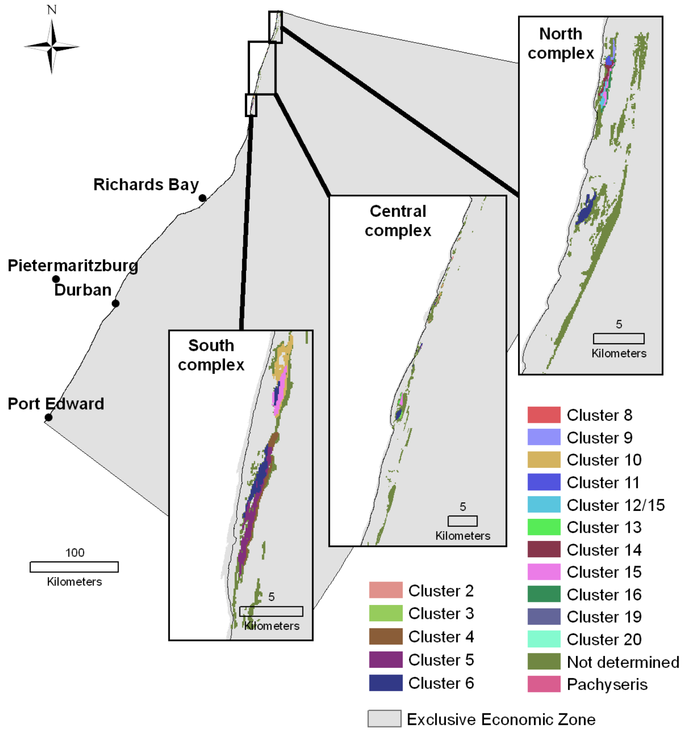

Fig 5. Spatial distribution of coral reefs in KwaZulu-Natal. Data provided by Schleyer and Celliers (2005).

Seventy-four estuaries were mapped as point elements along the coastline with the following attributes: name, average annual flow, open (yes or no) and irreplaceability value (Fig 6). The irreplaceability value was calculated in the framework of the KZN Estuaries Systematic Conservation Plan (Escott et al., unpublished). The area of influence of each estuary was mapped as a circular area around the estuary (Figure 6), and the radius of this circle was proportional to the average annual flow of the river and ranged between 500 m and 10 km (maximum value for the Tugela River). This distance was set according to discussions with regional experts and based on a regional study by [15].

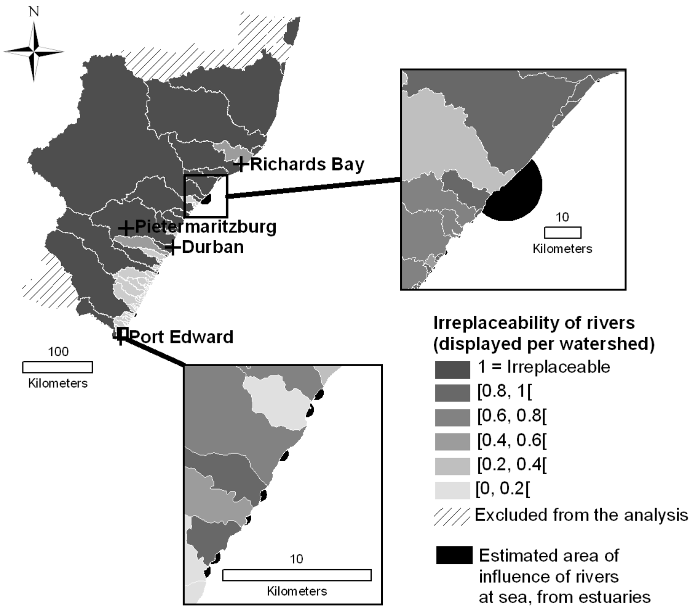

Fig 6. Marine area of influence estimated for each estuary in in KwaZulu-Natal.

2.2. Fish species distribution

The distribution of 81 fish species (including 14 shark species) was mapped at a resolution of 1 km by Philip Haupt [16]. A set of qualification criteria were set to select species whose distribution ranges were to be mapped in priority, from an estimated list of 1640 marine fish species as described in [17]. Three principles were used to identify these fish species: (i) limited conservation options, (ii) threatened species, and (iii) species that are inherently vulnerable. Seven characteristics (which pertain to one or more of the principles) were used to identify fish species for inclusion in Seaplan (endemic, conservation concern, life history vulnerability, higly resident, estuarine-dependant, rare or dependant on specialised habitats). The application of criterias provided a list of 280 species. Species for which fewer than 10 specimens have ever been recorded and/or species for which taxonomic uncertainty exists were removed from this list, leaving a total of 250 species. This final list was further reduced to a manageable list of 80 species.

IDRISI v16.5 was used to map the fish species distribution areas (referred to as FDA) by applying Boolean rules (suitable/non-suitable) to the following layers: depth, range, and distance to coast and habitat type (rocky/sandy/estuary). All these layers were then combined (multiplication operator) to model the FDA (see exemple of FDA map in Fig 7). The fractioning of FDA's into species life cycles areas (referred to as SLICES) was developped for a sub-group of 25 species based on available information. SLICES are spatial components associated with the following species life cycles: 1. Fish

Adults reproductive Areas including spawning/mating/pupping/gestating (elasmobranchs), 2. Juveniles feeding/growing areas and 3. Adults feeding/persisting areas.

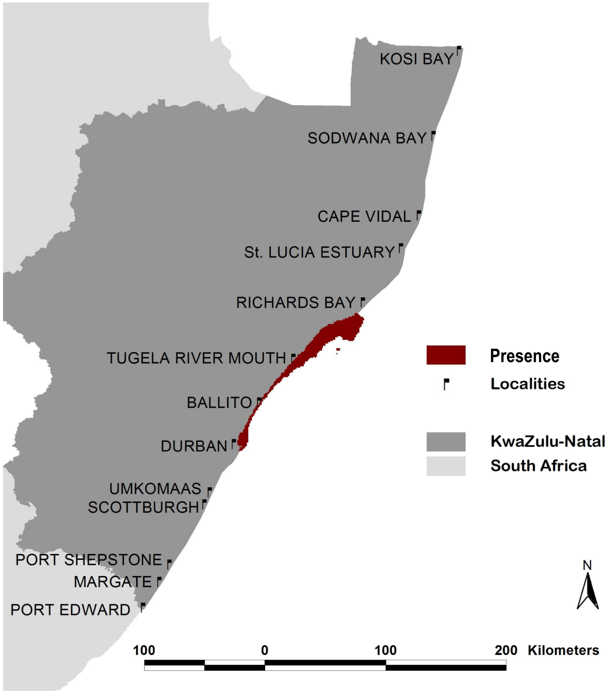

Fig 7. Presence-absence distribution of the squaretail kob (Argyrosomus thorpei) in KZN (from [16].

The fish distribution models and output maps were evaluated by a panel of 20 internationnally recognised south african experts identified during multiple independent workshops starting from December 2008. The maps were evaluated in a power point presentation including name, picture (photos supplied by Dennis King), distribution information and a map of the modelled distribution. Experts then drew on knowledge and experience to help to refine maps and provide more detailed information on core ranges and areas of special interest. The input from the experts was hugely valuable to fill in the knowledge gaps unavailable from the published literature. During the workshops, experts increasingly displayed confidence in the distribution models and the conservation planning exercise.

The distribution models were updated with the new information obtained in the workshops. The amended distribution models were then evaluated by Bruce Mann (ORI). Several of the shark distribution models were evaluated by Sheldon Dudley, Geremy Cliff and Sabine Wintner from KZN Sharks Board, who provided further useful information, especially in terms of species selection and new species on the IUCN Red List. Further information was supplied through email correspondence with several of the experts, in particular Bruce Mann and Sean Fennessy (ORI), the aforementioned

members from KZN Sharks board, and Allan Connell, throughout the duration of the project, which led to continuous updating of several of the species distribution models.

3. Fine-scale elements

3.1. Shoreline habitats

Fine-scale shoreline habitats for the entire Provincial coastline were mapped in 25 m stretches in the field, between the -2 m depth and the vegetation line within the following six zones (Fig 8, Fig 9) : supratidal (foredune and swash zone), intertidal (high-shore, mid-shore and low-shore) and infratidal (surf-zone). 27 different shoreline habitat types were mapped using those 6 zones. The intertidal has been classified in terms of structure, erosional state and nature of habitat provided for biological colonisation. Additionnal field sampling was conducted to obtain data to classify and map the morphodynamic types of beaches in KZN. Three major beach types were discerned: i) Reflective beaches, characterised by coarse sand and steep beach faces, associated with low biodiversity; ii) Intermediate beaches, have intermediate beach slopes with medium particle sizes and are often formed under exposed conditions; iii) Dissipative beaches, have wide surf zones, flat beach faces and fine sand. This latest beach type has the highest infaunal diversity, which is susceptible to human-induced disturbance. A detailed presentation of the shoreline habitat (and beaches types) mapping methods and results can be found in [1].

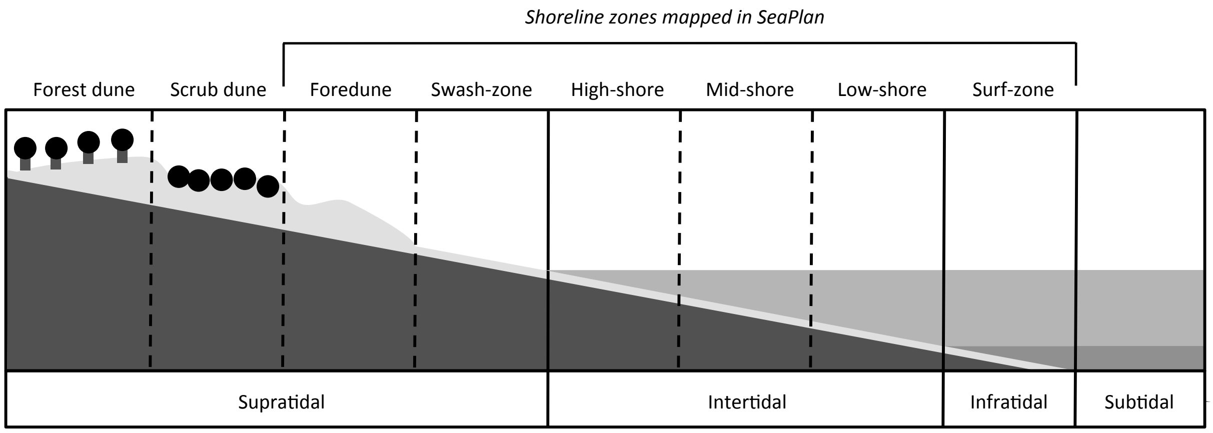

Fig 8. Spatial zonation of the shoreline and the 6 shoreline zones mapped in Seaplan. Those zone were used as the spatial containers of information on habitat types.

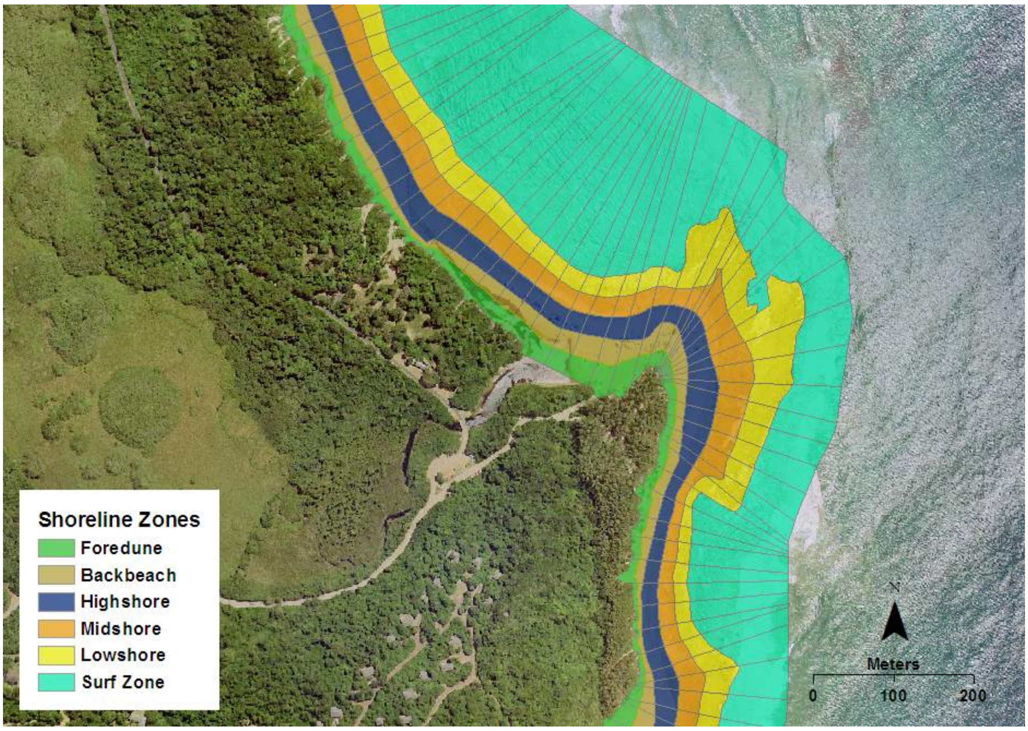

Fig 9. Image depicting the shoreline polygons. Habitat and transformation information can be queried for each polygon within the GIS database.

3.2. Fine-scale turtle nesting sites distribution

The two species included were the loggerhead turtle, Caretta caretta, and the leatherback, Dermochelys coriacea. In terms of the IUCN Red List, these two species were categorised as endangered and critically endangered, respectively. Both these species use the northern beaches of KZN as nesting areas which is an extremely vulnerable phase of their life cycles and thus the protection of these areas is essential.

The turtle monitoring program was started in 1965 by Dr George Hughes. Location of nesting females and hatchlings are recorded using turtle beacons established at approximately 500 m intervals by turtle monitors during the turtle season from November to March.

Owing to the wide ranging and complex nature of these species' movement patterns [13] it was not attempted to model their marine distributions. Both species are known to use the same areas as nesting sites [18] , [19] and therefore modelled nesting sites for both species were based on data from the Ezemvelo turtle monitoring programme. The final layer indicating their distribution range was approximated by the sandy shores habitat data in the shoreline habitat layer, up to the coastal dune, and stretching from Richards Bay in the south to the Mozambique border in the north of KZN [18]. Although more nesting sites are recorded in the northern parts of their nesting range compared to their southerly limit near Richards Bay, we did not take these differences into account for the

distribution models. This decision was based on lower data collection efforts outside of existing protected areas, in particular south of Cape Vidal, thus naturally biasing data towards the northern section of KZN and not accurately reflecting patterns south of the protected area, even though this is the end of their distribution range [19].

4. References

1. Harris JM, Livingstone T, Lombard AT, Lagabrielle E, Haupt P, Sink K, et al. Marine Systematic Conservation Assessment and Plan for KwaZulu-Natal - Spatial priorities for conservation of marine and coastal biodiversity in KwaZulu-Natal. Durban (South Africa): Ezemvelo KZN Wildlife Scientific Services. 2011. Technical Report.
2. Young P. An integrated marine GIS bathymetric dataset for KwaZulu-Natal [dissertation]. Durban: University of KwaZulu-Natal, South Africa; 2009.
3. GEBCO Task Group. User Guide to the Centenary Edition of the GEBCO Digital Atlas and its data sets. In: Jones MT, editor. GEBCO Sub-Committee on Digital Bathymetry. 2003.141 p.
4. Smith WHF, Sandwell DT. Global Sea Floor Topography from Satellite Altimetry and Ship Depth Soundings. Science. 1997;277(5334): 1956-1962.
5. Dingle RV, Goodlad SW, Martin AK. Bathymetry and stratigraphy of the northern Natal Valley (South West Indian Ocean): A preliminary account. Mar Geol. 1978;28: 89-106.
6. Birch GF. Quaternary sedimentation off the East Coast of Southern Africa (Cape Padrone to Cape Vidal). Bull Geol.Surv S. Afr. 1996; 118.
7. Weimerskirch H, Le Corre M, Jaquemet S, Potier M, Marsac F. Foraging strategy of a top predator in tropical waters: great frigatebirds in the Mozambique Channel. Mar Ecol Prog Ser. 2004;275: 297–308.
8. Castellani M. Identification of eddies from sea surface temperature maps with neural networks. Int J Remote Sens. 2006;27: 1601-1618.
9. Cayula J-F, Cornillon P. Edge detection algorithm for sst images. J Atmos Oceanic Technol. 1992;9: 67–80.
10. Backeberg BC, Johannessen JA, Bertino L, Reason CJ. The greater Agulhas Current system: An integrated study of its mesoscale variability. J Oper Oceanogr. 2008;1(1): 29-44.
11. Roberts JJ, Best BD, Dunn DC, Treml EA, Halpin PN. Marine Geospatial Ecology Tools: An integrated framework for ecological geoprocessing with ArcGIS, Python, R, MATLAB, and C++. Environ Model Softw. 2010;25(10): 1197-1207.
12. Findlay KP. The distribution of cetaceans off the coast of South Africa and South West Africa/Namibia [dissertation]. Pretoria (South Africa): University of Pretoria; 1989.

13. Miller WR, Ramsay PJ. South African Coalacanth Conservation and Genome Resource Programme – Multi-beam Geophysical Mapping of Coelacanth Habitats. Durban: South African Institute for Aquatic Biodiversity; 2002.
14. Ramsay PJ, Schleyer MH, Leuci R, Muller GA, Celliers L, Harris JM, et al. The development of an expert marine geographical information system to provide an environmental and economic decision-support system for coastal tourism and leisure developments within the Lubombo Spatial Development Initiative. Pretoria: South African Department of Arts, Culture; 2006. Science and Technology of South Africa Innovation Fund Project–24401.
15. Cooper JAG. The role of extreme floods in estuary-coastal behaviour: contrasts between river- and tide-dominated microtidal estuaries. Sediment Geol. 2002;150: 123-137.
16. Haupt P. The use of fish species in a marine conservation plan for KwaZulu-Natal. [dissertation]. Durban (South Africa): University of Kwazulu Natal; 2011.
17. Junor FJR. Importance ratings for Marine Fish of Natal. Document 1, Phase 1, of programme to prevent man-induced extinction of indigenous species of Natal. Pietermaritzburg (South Africa): Natal Parks Board; 1992.
18. Nel R. Status of leatherback turtles in South Africa. In: Hamann M, Limpus C, Hughes G, Mortimer J, Pilcher N, editors. Assessment of the conservation status of the leatherback turtle in the Indian Ocean and South East Asia. Bangkok, Thailand: IOSEA; 2006. p. 125-130.
19. Bachoo S, Olbers J. Ezemvelo KZN Wildlife turtle monitoring research. Haupt PW, editor. Durban (South Africa); 2009.
