# Supplementary material for: Multi-scale multi-level marine spatial planning: A novel methodological approach applied in South Africa
Source: PLoS One. 2018 Jul 3;13(7):e0192582. doi: 10.1371/journal.pone.0192582 (PMC6029778; doi:10.1371/journal.pone.0192582)
Supplement: S2 Appendix — (PDF) [file pone.0192582.s002.pdf]

S2 Appendix. Data and methods for mapping human activities and the index of anthropogenic pressures in the SeaPlan planning domain

Adapted from [1].

1. Overview

We define drivers of change as those human activities that impact marine ecosystem structure and function negatively. Data on the spatial extent and intensity of drivers of change were combined into an index of cumulative anthropogenic pressures map, which used an expert-driven weighting approach to assign higher values to pressures with known high impacts (e.g. trawling), and lower values to pressures with lower impacts (e.g. scuba diving). The cumulative impact map was then used to assign a cost value to each planning unit for the conservation planning analyses conducted with MARXAN software [2]. This method assumes that high cost values are a proxy for actual pressure, as well for areas where high socio-economic costs would be incurred in the event of conservation management measures being instituted.

2. List of human activities

We developed a list of broadly-defined human activities, both marine and land-based, that threaten marine ecosystems within the EEZ of the KwaZulu-Natal (KZN) Province (Table 1, column "Human activity"). Available data were sourced to map the extent of a subset of these activities as presence/absence distribution data, and where possible, we modelled intensity (Table 1, columns "Geographic proxy" and "Distribution data type"). Workshops with managers, scientists and marine resource users informed this process.

Table 1. Human activities in the marine environment in the SeaPlan planning domain. In the column "Data type", P stands for presence/absence data, I stands for intensity data.

	Human activity	Geographic proxy	Data type	Weight
1	Commercial linefishing	Commercial linefishing area	P	16
2	Commercial pelagic longlining	Pelagic longlining area	I	20
3	Commercial inshore crustacean trawling	Crustacean trawling area	I	21.5
4	Commercial offshore crustacean trawling			
5	Commercial deepwater rocklobster trap fishing			
6	Commercial rock oyster harvesting	Commercial oyster harvesting area	P	19
7	Small scale seine-net fishery for sardines	Small-scale commercial seine netting of sardines area	P	5
8	Small scale experimental fishery for redeye			
9	Small scale seine-net fishery (Durban)	Small-scale seine netters (Durban) area	P	7
10	Subsistence linefishing	Subsistence linefishing area	P	7
11	Subsistence rocky-shore intertidal benthic invertebrate harvesting	Subsistence intertidal invertebrate extraction area	P	6
12	Unpermitted subsistence intertidal harvesting	Unpermitted subsistence intertidal harvesting area	P	15
13	Subsistence- sandy-beach invertebrate harvesting			
14	Recreational linefishing (shore fishing)	Shore angling area	I	10
15	Recreational linefishing (skiboat gamefishing)	Recreational linefishing area (skiboat gamefishing)	I	5
16	Recreational linefishing (skiboat bottomfishing)	Recreational linefishing area (skiboat bottomfishing)	I	12
17	Recreational paddle-ski (gamefishing)	Recreational paddle-ski linefishing area (gamefish)	I	6
18	Recreational paddle-ski (bottomfishing)	Recreational paddle-ski linefishing area (bottomfish)	I	6
19	Recreational spearfishing (gamefish)	Recreational spearfishing area (gamefish)	I	6
20	Recreational spearfishing (bottomfish)	Recreational spearfishing (bottomfish)	I	8
21	Recreational spearfishing	Recreational spearfishing	P	5
22	Recreational charter boat linefishing	Recreational charter boat fishing	P	15
23	Recreational east coast rock lobster harvesting	Recreational east coast rock lobster harvesting	P	5
24	Recreational mussel harvesting	Recreational mussel harvesting	P	6
25	Recreational oyster harvesting	Recreational oyster harvesting	P	8
26	Recreational octopus harvesting	Recreational octopus harvesting	P	5
27	Recreational bait fishing (cast-netting)			
28	Recreational bait harvesting - rocky shore other invertebrates (limpets, redbait, rock crabs, etc.)	Recreational bait fishery (rocky shores) collection	P	6
29	Recreational bait harvesting - sandy beach other invertebrates (mole crabs, ghost crabs etc.)	Recreational bait fishery (sandy beach) collection	P	5
30	Shark Control Programme	Area of influence of shark-nets along KZN coast	P	18
31	Aquarium collecting – commercial			
32	Aquarium collecting – recreational	Recreational aquarium collection	P	7
33	Ornamental shell collecting			
34	Mariculture – prawn farming (Amatikulu)			
35	Alien invasive species	Potential distribution of alien species	P	19
36	Sand winning			
37	Fossil fuel mining			

38	Titanium mining			
39	Shipping general	Shipping lanes	I	16
40	Shipping casualties			
41	Effluent pipelines - industrial waste	Area of influence of industrial pipelines	I	18
42	Outfalls - sewerage and stormwater	Residential sewage pipelines	P	9.5
43	Poor catchment management			
44	Poor estuary management			
45	Boat launch sites - without hard structures			
46	Off road vehicles - beach driving	Permitted beach driving area	P	8
47	Concession driving areas	Permitted beach driving area for turtle tourism	P	8
48	Land-based marine ecotourism - e.g. turtles			
49	Boat-based ecotourism - e.g. whales, dolphins			
50	Diver based 1 e.g. scuba diving	Scuba diving area	I	5
51	Diver based 1 e.g. deep diving	Coelacanth deep diving area	I	5
52	Diver based 2 e.g. shark diving			
53	Coastal infrastructure	Area of influence of urban areas	I	19
54	Boat launch sites – with hard structures			
55	Harbours	Area of influence of harbours	P	19
56	Jet-skis	Jet-skis area	Yes	5
57	Shoreline frequentation (accessibility to people)	Shoreline frequentation (accessibility to people)	Yes	5
58	Climate change impacts			

3. Geographic Information System (GIS) mapping methods

The spatial extents of a subset of 38 pressures were mapped as presence/absence GIS layers (Table 1). These extents were defined by experts and were based on the following parameters: distance from the coast, depth, range of vessel, and legal restrictions (for instance, prohibited activities such as fishing in certain zones of a MPA). All layers were rasterised with a spatial resolution of 5 m. Fig 1 shows some examples of presence/absence maps. More details are available in [1].

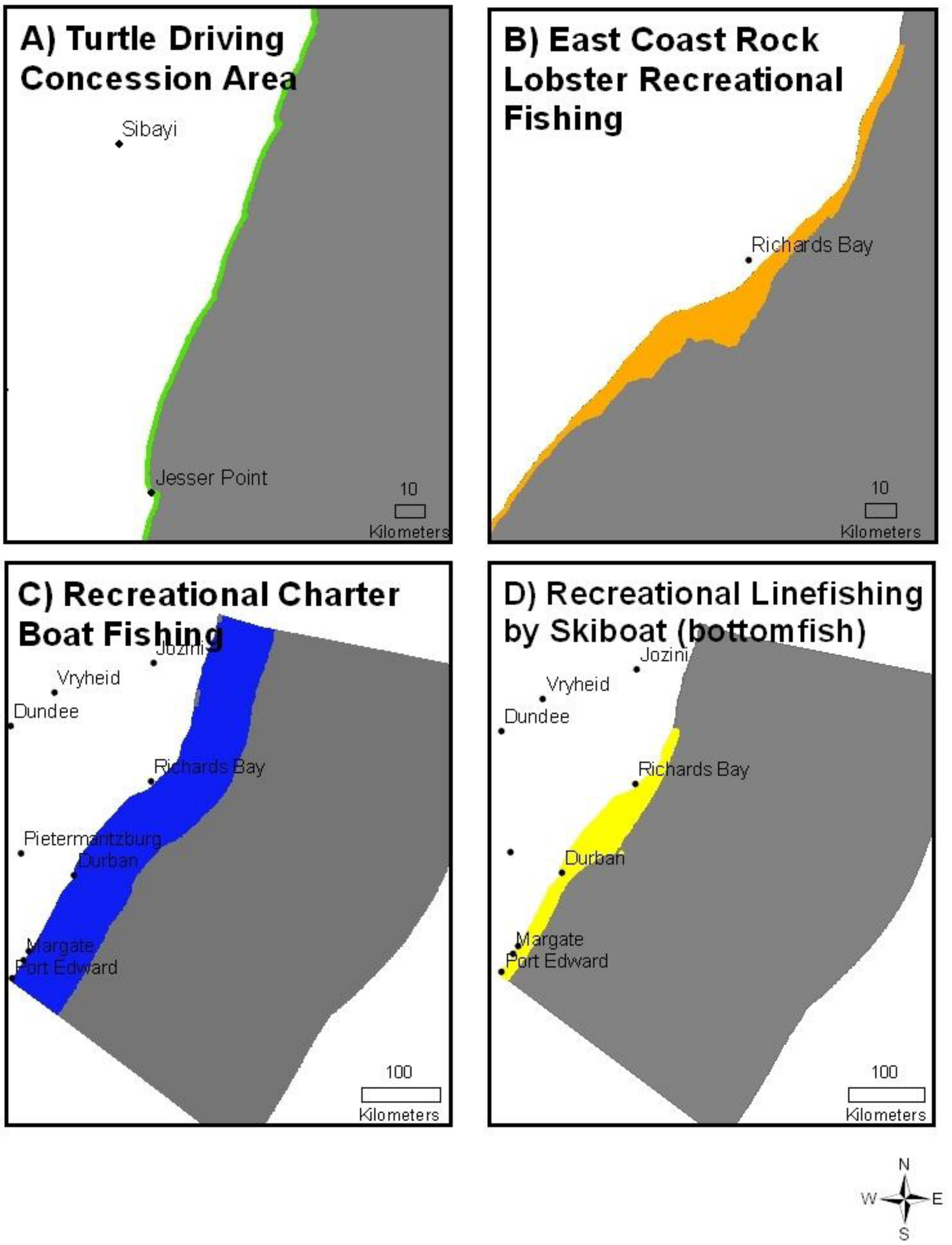

Fig 1. The spatial extents of four pressures in the SeaPlan planning domain. A) Turtle driving concessions areas; B) East Coast Rock Lobster recreational fishing areas; C) Recreational charter boat fishing areas; D) Recreational line fishing by ski boats for bottom fish. Colours indicate areas where these activities may take place, as opposed to areas of actual distribution.

Continuous intensity layers (from low to high intensity) were generated for 16 pressures (Table 1). Various modelling techniques including spatial interpolation, fuzzy logic and kernel analysis were used (see section 6 below).

4. Weighting pressures

In the absence of quantitative data on the actual impacts of different pressures on different components of marine ecosystems, we assigned weights to pressures using the method outlined in Table 2. Impact values were determined by an expert workshop. Values from each of the seven different ecosystem impact types listed in Table 2 were summed per pressure, and total impact values are listed in Table 1. These total impact values were used to weight pressure layers when they were combined into a cumulative pressure map.

Table 1. Impact values assigned to different pressures, depending on their ecosystem impacts (some examples are listed in the left hand column).

	Ecosystem impacts	Impact value range (low - high)	Impact value – and type of pressure (examples)
1	Impact on species	1-5	1- Seine netting for sardines<br>5- Prawn trawling
2	Impact in terms of biomass	1-5	1- Subsistence fishing,<br>5- Commercial longlining
3	Habitat destruction	1-5	1- Scuba diving<br>5- Demersal crustacean trawl fishing
4	Trophic impacts	1-3	1- Octopus fishing<br>3- Commercial longlining
5	Risk (large, but infrequent)	1-3	1- Recreational skiboat fishing.<br>3- Oil spills (shipping lane)
6	Habitat degradation	1-3	1- Sandy beaches bait collection<br>3- Sewage pipelines
7	Economic Incentive	1-3	1- Recreational linefishing<br>3- Commercial linefishing

5. Cumulative pressure map

Presence/absence pressures layers held values of one or zero, and intensity pressure layers were scaled from zero to one. The total impact value from Table 1 was used as a multiplier for each pressure layer, after which all pressure layers were summed into one final cumulative pressure map (Fig 2). These final pressure values were used to calculate the cost values for the Marxan analyses, where cost per planning unit = mean of final pressure values in the planning unit. Costs were calculated for each of the planning unit layers all three levels of resolution. High cost values depict areas to be avoided when identifying priority areas for protection.

Highest pressures occur closer to the coast (particularly around launching sites) and increase southwards from Richards Bay to Durban and remain high along the coastline down to Port Edward. These areas are the economic centres of the KZN coast. Areas of high pressure are also found offshore as a result of pelagic longlining, and the shipping lane linking Durban with East Asia.

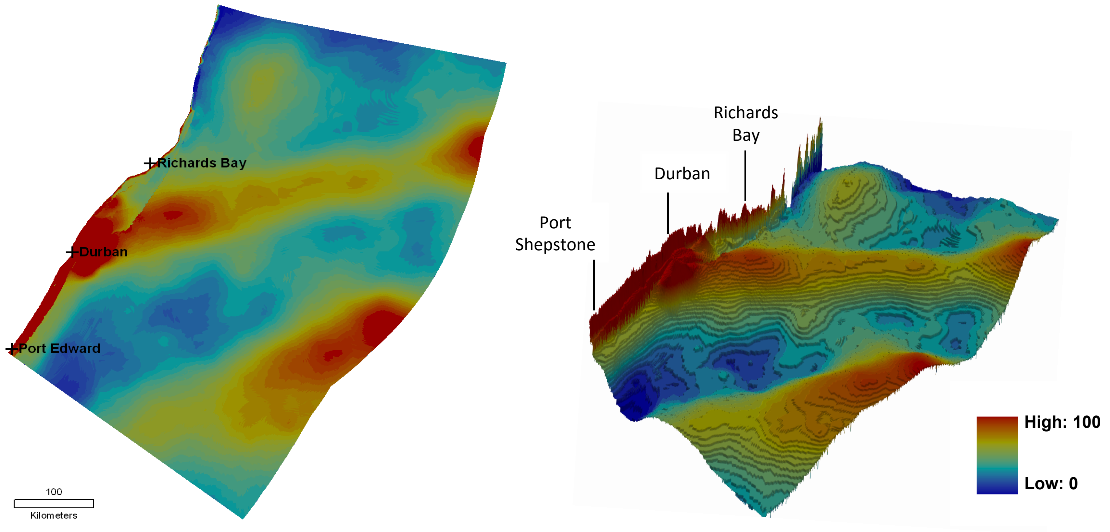

Fig 2. 2D and 3D representation of the cumulative pressure map for the SeaPlan planning domain.

6. Intensity mapping methods

6.1 Small vessel boating activities

The method described below was used to model threats No 15, 16, 17, 18, 19, 20, 50, 51, 56 (**Erreur ! Source du renvoi introuvable.**).

The intensity of small vessel (< 10 m length) boating activities was derived from an initial map of launch sites, to which data on launch site statistics was attached. Data were obtained from the Oceanographic Research Institute (ORI) (i.e. the Boat Launch Site Monitoring System) and consisted of records on the number and type of boats (i.e. ski-boats, inflatable boats, paddle-skis and jet-skis) launched over a one year period (2006) from the participating launch sites along the KZN coastline. These launch sites were further grouped into 40 sites. A 100 m grid was derived for each boat type (n = 4), with each launch site being represented by a single cell grid. The grid cell value was equal to the number of boats launched over a year period. The grids were log(X+1) transformed in order to standardise the values. This layer is referred to as [Layer 1]. Note that for diving-related activities, it was considered that 70% of divers dived from inflatable boats and 30% from ski boats.

The potential spatial distribution of boat fleets, per boat type, from each launch site was modelled using the set of fuzzy rules described in Fig 3. This set of rules was developed with experts: B. Mann (ORI), J. Harris (Ezemvelo KZN Wildlife) and N. Scott-Williams (Subtech Diving). It was implemented in ArcGis 9.2 software using the toolbox “Spatial Data Modeller”.

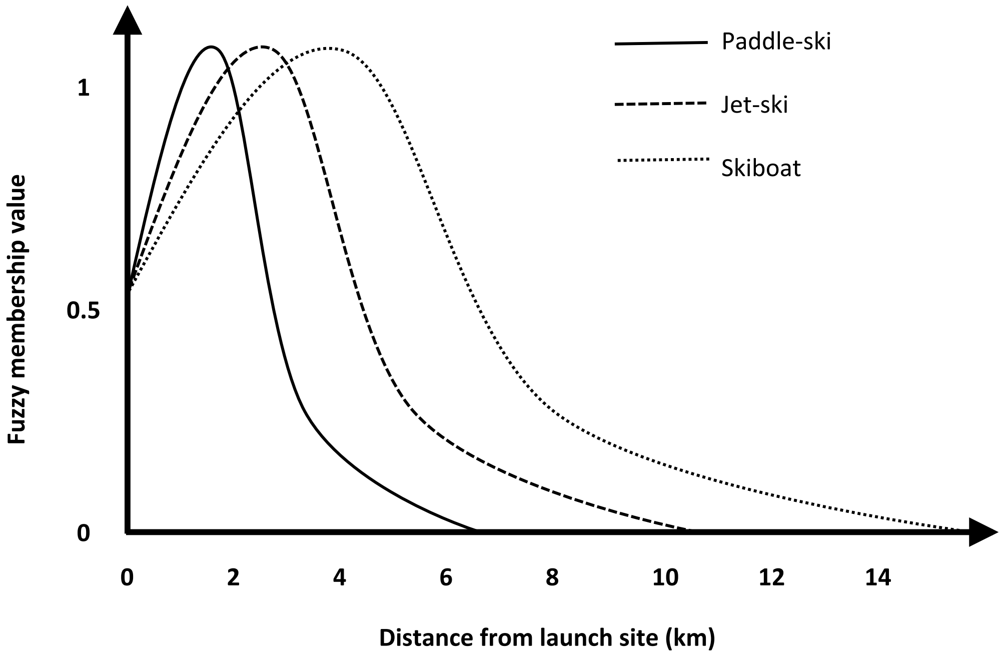

Fig 3. Fuzzy function showing membership to highly frequented areas, per boat type, depending on distance from launch sites.

Technical/logistical constraints limit the geographic distribution of the boat fleet and a number of assumptions were made based on existing knowledge. For example, skiboats are limited by engine, fuel, time and safety constraints and thus the assumption is that they do not travel more than 50 km from their launch site. Based on this set of rules, a probable distribution of boats, per boat type, from each launch site, was derived. Other more complicated parameters, such as preferences for fishing spots were not integrated in the analysis. The final fuzzy layer contains values ranging from 0 to 1 and it is referred to as [Layer 2].

A kernel analysis (sum operator, circular shape) was performed on [Layer 1] to assess the number of boats that could potentially reach each cell in the EEZ. The radius of this focal analysis was set for each boat type (n=4), according to their maximum operational distance. Thus if a cell can be reached from an harbour H1 with 20 launches per year and from an harbour H2 with 30 launches per year,

then this cell received the value 20 + 30 = 50. The layer resulting from the focal analysis was transformed to a fuzzy layer with values ranging from 0 to 1, hereafter referred to as [Layer 3].

Finally, [Layer 2] and [Layer 3] were combined using a fuzzy sum operator. The resulting layer is the density of boats, per boat type in the ocean. It is assumed that boat density acts as a surrogate for fishing pressure (given that most boats in the analysis were fishing).

6.2 Industrial pipelines

Five industrial pipelines were identified along the KZN coastline using GPS coordinates obtained from experts or the industry itself. The discharge of each pipeline was determined and mapped as a grid layer (100 m resolution) with one cell for each pipeline. The value of the cell was equal to the discharge. This layer is referred to as [Layer 1]. Expert advice provided by T. Samaai (Department of Environment Affairs) established the maximum distance of contamination of a pipeline at 5 km, with a minimum distance of 2 km. Based on this information, the maximum area of influence for each pipeline was assumed (
Table 3). In a fourth step a kernel analysis (sum operator, circular shape) was applied to [Layer 1]. The radius of the kernel was the maximum area of influence of the pipeline. This layer was transformed to fuzzy values ranging from 0 to 1, hereafter referred to as [Layer 2]. Distances to the pipelines were calculated as a grid layer. This layer was transformed to fuzzy values with a linearly decreasing function with a value of 1 at the pipeline down to 0 outside the area of influence. This layer is referred to as [Layer 3]. Finally [Layer 2] and [Layer 3] were combined using a fuzzy sum operator. The resulting layer is the modelled intensity of the impact of industrial pipelines.

Table 3. Data and data sources for industrial pipelines.

Name	Source	Length (km)	Discharge (m^3/hr)	Radius (km)
End Cap -AECI	Caroline Dickens - AECI	6.5	45	2
Huntsman Tioxide - I	Irene -SA Tioxide	1.7	30	2
Central Works Outfall	Tim McClurg-CSIR	3.2	60	2
Southern Works Outfall	Tim McClurg-CSIR	4.2	50	2
SAPPI SAICCOR	Derek Airey-SAPPI	6.5	45	4
Richards Bay	SAN chart 1993.47	4.95	30	3

6.3 Influence of urban areas

The urban layer was extracted from the KZN landcover data [4]. Distances to core urban areas were extracted and mapped as a grid layer. This grid layer was further transformed into a fuzzy layer using a linearly decreasing function. This function was set as follows: at 0 km the fuzzy value is 1 (highly impacted) and further than 5 km the fuzzy value is 0 (no impact).

6.4 Shipping lanes

The dataset on shipping lanes was extracted from the study by [5]. Data were collected over 12 months from the beginning of October 2004 (collected as part of the World Meteorological Organization Voluntary Observing Ships Scheme (VOS); http://www.vos.noaa.gov/vos_scheme.shtml). Mobile ship data were connected and used to create ship tracks, under the assumption that ships travel in straight lines. The 799 853 line segments were then buffered to be 1 km wide thus accounting for the width of shipping lanes, and all buffered line segments were summed to account for overlapping ship tracks. The summed ship tracks were converted to raster data. This produced 1 km^2 raster cells with values ranging from 0 to 1158, the maximum number of ship tracks recorded in a single 1 km^2 cell. Because the VOS program is voluntary, it should be noted that the estimates on the impact of shipping are biased (in an unknown way) to locations and types of ships engaged in the program.

6.5 Crustacean trawl and pelagic longlining

Data on crustacean trawling and pelagic longlining were provided by K. Sink (South African National Biodiversity Institute). Data were provided in 16x16 km square grid cells. The intensity of these activities was mapped using the total number of trawling hours for crustacean trawling and the catch per unit effort (CPUE) for pelagic longlining. Data was then interpolated to fit a 100 m resolution grid using a Kriging technique (with 20 surrounding points).

6.6 Shore angling

Data on shore angling were provided by B. Mann (ORI) who conducted an aerial survey of the KwaZulu-Natal coast to determine total shore angling effort [6]. Between March 2007 and February 2008, 36 flights were undertaken, half along the north coast (Virginia to Kosi Bay) and half along the south (Virginia to Port St Johns). The plane flew at a low level (400-500 ft) along the coastline at a speed of 70-90 knots, counting all observed shore anglers using a manual tally counter. The number of anglers per km of coast was determined for each section of the coast. Spatial data were further transformed to a fuzzy layer using a linear function, with final values ranging from 0 to 1.

6.7 Shoreline frequentation (accessibility to people)

Different types of shoreline access were noted during the coastal mapping exercise and recorded in the shoreline habitat layer (e.g. parking lots and piers; toll roads; public access paths, viewing platforms, promenades, stairways, etc.). These access areas were weighted from 1 (low frequentation) to 5 (high frequentation). Ezemvelo KZN Wildlife experts determined the maximum walking distance from these points to be approximately 1 km with the majority of people found within a 200 m radius of the access point. This rule was applied using a fuzzy logic approach based on distance to access points. A frequentation layer was produced for each access category and the final five layers were weight summed. The resulting layer was linearly transformed into a fuzzy layer with values ranging from 1 (highly frequented) to 0 (not frequented).

7. References

1. Harris JM, Livingstone T, Lombard AT, Lagabrielle E, Haupt P, Sink K, et al. Marine Systematic Conservation Assessment and Plan for KwaZulu-Natal - Spatial priorities for conservation of marine and coastal biodiversity in KwaZulu-Natal. Durban (South Africa): Ezemvelo KZN Wildlife Scientific Services. 2011. Technical Report.
2. Ball I, Possingham H. MARXAN (V1. 8.2). Marine Reserve Design Using Spatially Explicit Annealing, a Manual. Brisbane: University of Queensland; 2000. 69 p.
3. ESRI. ArcGIS Desktop: Release 10. Redlands (CA): Environmental Systems Research Institute; 2011.
4. Jewitt D, Goodman PS, Erasmus BFN, O'Connor TG, Witkowski ETF. Systematic land-cover change in KwaZulu-Natal, South Africa: Implications for biodiversity. S Afr J Sci. 2015; 111(9/10): Art. #2015-0019. doi: http://dx.doi.org/10.17159/ sajs.2015/20150019
5. Halpern BS, Walbridge S, Selkoe KA, Kappel CV, Micheli F, D'Agrosa C, et al. Global Map of Human Impact on Marine Ecosystems. Science. 2008; 319: 948-952.
6. Mann BQ, Nanni G, Pradervand P. A monthly aerial survey of the KwaZulu-Natal marine shore fishery. Durban (South Africa): Oceanographic Research Institute; 2008. Report No.: 264.
