# Supplementary material for: Multi-scale multi-level marine spatial planning: A novel methodological approach applied in South Africa
Source: PLoS One. 2018 Jul 3;13(7):e0192582. doi: 10.1371/journal.pone.0192582 (PMC6029778; doi:10.1371/journal.pone.0192582)
Supplement: S3 Appendix — (PDF) [file pone.0192582.s003.pdf]

S3 Appendix. List of biodiversity elements and conservation targets

Code	Category of elements	Biodiversity elements	Surface (km sq.)	Area in MPA type A (%)	Area in MPA type B (%)	Area in MPA type C (%)	Area without protection (%)	A Target (%)	B Target (%)	% of A target achieved	% of B target achieved
1100003	Broad-scale habitat units - Shelf (-30m - - 200m)	A3 - Turbid Tugela	1711,870	0%	0%	0%	100%	10%	10%	0%	0%
1100004	Broad-scale habitat units - Shelf (-30m - - 200m)	A4 - Sandy shelf	3856,158	0%	0%	1%	99%	10%	10%	0%	0%
1100005	Broad-scale habitat units - Shelf (-30m - - 200m)	A5 - Muddy shelf	1895,477	0%	0%	0%	100%	10%	10%	0%	0%
1100006	Broad-scale habitat units - Shelf (-30m - - 200m)	A6 - Sloping shelf	2068,397	0%	0%	0%	100%	10%	10%	0%	0%
1100007	Broad-scale habitat units - Shelf (-30m - - 200m)	B1 - Steep slope	10216,531	2%	2%	2%	94%	10%	10%	16%	18%
1100008	Broad-scale habitat units - Deep (< - 200m)	B2 - Agulhas current	33788,269	0%	0%	0%	100%	10%	10%	0%	0%
1100009	Broad-scale habitat units - Deep (< - 200m)	B3 - Agulhas eddy	31963,337	0%	0%	0%	100%	10%	10%	0%	0%
1100010	Broad-scale habitat units - Deep (< - 200m)	B4 - Deep Agulhas	35724,720	0%	0%	0%	100%	10%	10%	0%	0%
1100011	Broad-scale habitat units - Deep (< - 200m)	C1 - Deep offshore	48115,533	0%	0%	0%	100%	10%	10%	0%	0%
1100012	Broad-scale habitat units - Deep (< - 200m)	C2 - Deep Eddy transition	62570,257	0%	0%	0%	100%	10%	10%	0%	0%
3100001	Semi-permanent eddies	Semi-permanent eddies	27556,228	0%	0%	0%	100%	10%	10%	0%	0%
3300001	Semi-permanent sea surface temperature fronts	Semi-permanent sea surface temperature fronts	2905,425	0%	0%	0%	100%	10%	10%	0%	0%
3500001	Semi-permanent chlorophyll-a fronts	Semi-permanent chlorophyll-a fronts	551,691	0%	0%	0%	100%	10%	10%	0%	0%
1410001	Rocky Reefs - Nearshore (0 - -30 m)	Rocky reefs in Broad-scale habitat units A1 - Delagoa (0 - -10 m)	45,884	0%	0%	4%	96%	10%	10%	0%	0%
1420001	Rocky Reefs - Nearshore (0 - -30 m)	Rocky reefs in Broad-scale habitat units A1 - Delagoa (-10 - -30 m)	239,586	0%	1%	10%	90%	10%	10%	0%	7%
1410002	Rocky Reefs - Nearshore (0 - -30 m)	Rocky reefs in Broad-scale habitat units A2 - Natal (0 - -10 m)	16,111	22%	30%	48%	0%	10%	10%	100%	100%
1420002	Rocky Reefs - Nearshore (0 - -30 m)	Rocky reefs in Broad-scale habitat units A2 - Natal (-10 - -30 m)	50,442	33%	34%	33%	0%	10%	10%	100%	100%
1430001	Rocky Reefs - Shelf (-30m - -200m)	Rocky reefs in Broad-scale habitat units A3 - Turbid Tugela	41,986	0%	0%	0%	100%	10%	10%	0%	0%
1430002	Rocky Reefs - Shelf (-30m - -200m)	Rocky reefs in Broad-scale habitat units A4 - Sandy shelf	650,216	0%	0%	4%	96%	10%	10%	0%	0%
1430003	Rocky Reefs - Shelf (-30m - -200m)	Rocky reefs in Broad-scale habitat units A5 - Muddy shelf	52,910	0%	0%	0%	100%	10%	10%	0%	0%

Code	Category of elements	Biodiversity elements	Surface (km sq.)	Area in MPA type A (%)	Area in MPA type B (%)	Area in MPA type C (%)	Area without protection (%)	A Target (%)	B Target (%)	% of A target achieved	% of B target achieved
1430004	Rocky Reefs - Shelf (-30m - -200m)	Rocky reefs in Broad-scale habitat units A6 - Sloping shelf	19,449	0%	0%	0%	100%	10%	10%	0%	0%
1430005	Rocky Reefs - Shelf (-30m - -200m)	Rocky reefs in Broad-scale habitat units B1 - Steep slope	87,491	18%	24%	39%	19%	10%	10%	100%	100%
1711043	Canyons	Canyon Delagoa ID 43	3,282	59%	0%	41%	0%	0%	100%	na	59%
1711048	Canyons	Canyon Delagoa ID 48	2,755	0%	0%	100%	0%	100%	0%	0%	na
1712002	Canyons	Canyon Natal ID 2	3,404	0%	0%	0%	100%	100%	0%	0%	na
1712006	Canyons	Canyon Natal ID 6	16,787	0%	0%	0%	100%	0%	100%	na	0%
1510005	Coral reefs (North complex)	Cluster 6 North Complex	1,430	100%	0%	0%	0%	40%	40%	100%	100%
1510017	Coral reefs (North complex)	Deep cluster 6 North Complex	0,685	100%	0%	0%	0%	40%	40%	100%	100%
1510007	Coral reefs (North complex)	Cluster 9 North Complex	0,437	100%	0%	0%	0%	40%	40%	100%	100%
1510009	Coral reefs (North complex)	Cluster 11 North Complex	0,395	100%	0%	0%	0%	80%	20%	100%	100%
1520012	Coral reefs (North complex)	Cluster 12 and 15 North Complex	0,925	100%	0%	0%	0%	40%	40%	100%	100%
1510013	Coral reefs (North complex)	Cluster 14 North Complex	0,694	100%	0%	0%	0%	50%	30%	100%	100%
1510010	Coral reefs (North complex)	Cluster 15 North Complex	0,645	100%	0%	0%	0%	80%	20%	100%	100%
1510014	Coral reefs (North complex)	Cluster 16 North Complex	0,928	100%	0%	0%	0%	50%	30%	100%	100%
1510018	Coral reefs (North complex)	Not determined North Complex	29,434	74%	26%	0%	0%	40%	40%	100%	100%
1520001	Coral reefs (Central complex)	Cluster 2 Central Complex	0,511	0%	100%	0%	0%	80%	20%	0%	100%
1520002	Coral reefs (Central complex)	Cluster 3 Central Complex	0,195	0%	0%	100%	0%	40%	40%	0%	0%
1520005	Coral reefs (Central complex)	Cluster 6 Central Complex	1,550	0%	25%	75%	0%	40%	40%	0%	63%
1520006	Coral reefs (Central complex)	Cluster 8 Central Complex	0,114	0%	100%	0%	0%	40%	40%	0%	100%
1520008	Coral reefs (Central complex)	Cluster 10 Central Complex	0,170	0%	100%	0%	0%	40%	40%	0%	100%
1520011	Coral reefs (Central complex)	Cluster 13 Central Complex	0,545	0%	0%	100%	0%	50%	30%	0%	0%
1510012	Coral reefs (Central complex)	Cluster 14 Central Complex	0,038	0%	100%	0%	0%	40%	40%	0%	100%
1520013	Coral reefs (Central complex)	Cluster 15 Central Complex	0,770	0%	6%	94%	0%	50%	30%	0%	19%
1520016	Coral reefs (Central complex)	Cluster 20 Central Complex	0,004	0%	100%	0%	0%	100%	0%	0%	na
1520019	Coral reefs (Central complex)	Pachyseris Central Complex	0,030	0%	0%	100%	0%	80%	20%	0%	0%
1520018	Coral reefs (Central complex)	Not determined Central Complex	20,189	0%	29%	71%	0%	40%	40%	0%	71%

Code	Category of elements	Biodiversity elements	Surface (km sq.)	Area in MPA type A (%)	Area in MPA type B (%)	Area in MPA type C (%)	Area without protection (%)	A Target (%)	B Target (%)	% of A target achieved	% of B target achieved
1530003	Coral reefs (South complex)	Cluster 4 South Complex	1,958	100%	0%	0%	0%	40%	40%	100%	100%
1530004	Coral reefs (South complex)	Cluster 5 South Complex	3,882	100%	0%	0%	0%	40%	40%	100%	100%
1530005	Coral reefs (South complex)	Cluster 6 South Complex	2,658	100%	0%	0%	0%	40%	40%	100%	100%
1530008	Coral reefs (South complex)	Cluster 10 South Complex	2,852	100%	0%	0%	0%	40%	40%	100%	100%
1530013	Coral reefs (South complex)	Cluster 15 South Complex	1,560	100%	0%	0%	0%	50%	30%	100%	100%
1530015	Coral reefs (South complex)	Cluster 19 South Complex	0,018	100%	0%	0%	0%	100%	0%	100%	na
1530018	Coral reefs (South complex)	Not determined South Complex	9,004	94%	0%	6%	0%	40%	40%	100%	100%
1210049	Fine-scale shoreline habitat units - Foredune	Rock boulders in foredune	0,054	0%	0%	36%	64%	0%	10%	na	0%
1210051	Fine-scale shoreline habitat units - Foredune	Broken rocks in foredune	0,005	100%	0%	0%	0%	0%	10%	na	100%
1210056	Fine-scale shoreline habitat units - Foredune	Rock Broken Ledge High in foredune	0,001	0%	0%	100%	0%	0%	10%	na	0%
1210059	Fine-scale shoreline habitat units - Foredune	Scattered rocks in foredune	0,000	0%	0%	0%	100%	0%	10%	na	0%
1210060	Fine-scale shoreline habitat units - Foredune	Solid Continuous Rocks in foredune	0,006	0%	0%	19%	81%	0%	10%	na	0%
1210053	Fine-scale shoreline habitat units - Foredune	High rock ledge in foredune	0,012	0%	0%	52%	48%	0%	10%	na	0%
1220049	Fine-scale shoreline habitat units - Swash-zone	Rock boulders in topshore	0,120	0%	1%	20%	79%	0%	20%	na	3%
1220051	Fine-scale shoreline habitat units - Swash-zone	Broken rocks in topshore	0,116	45%	9%	3%	43%	0%	20%	na	100%
1220053	Fine-scale shoreline habitat units - Swash-zone	High rock ledge in topshore	0,059	7%	1%	0%	92%	0%	20%	na	41%
1220054	Fine-scale shoreline habitat units - Swash-zone	Low rock ledge in topshore	0,014	0%	0%	28%	72%	0%	20%	na	0%
1220056	Fine-scale shoreline habitat units - Swash-zone	Rock Broken Ledge High in topshore	0,033	0%	0%	13%	87%	0%	20%	na	0%
1220057	Fine-scale shoreline habitat units - Swash-zone	Rock Broken Ledge Low in topshore	0,002	0%	0%	25%	75%	0%	20%	na	0%
1220059	Fine-scale shoreline habitat units - Swash-zone	Scattered rocks in topshore	0,162	29%	5%	3%	63%	0%	20%	na	100%
1220060	Fine-scale shoreline habitat units - Swash-zone	Solid Continuous Rocks in Swash-zone	0,213	0%	0%	10%	90%	0%	20%	na	0%
1230060	Fine-scale shoreline habitat units - High-shore intertidal zone	Solid Continuous Rocks in High-shore	0,314	0%	0%	23%	77%	10%	10%	4%	0%

Code	Category of elements	Biodiversity elements	Surface (km sq.)	Area in MPA type A (%)	Area in MPA type B (%)	Area in MPA type C (%)	Area without protection (%)	A Target (%)	B Target (%)	% of A target achieved	% of B target achieved
1230049	Fine-scale shoreline habitat units - High-shore intertidal zone	Rock boulders in High-shore	0,128	0%	4%	16%	80%	0%	20%	na	19%
1230051	Fine-scale shoreline habitat units - High-shore intertidal zone	Broken rocks in High-shore	0,084	2%	5%	16%	77%	10%	10%	24%	48%
1230053	Fine-scale shoreline habitat units - High-shore intertidal zone	High rock ledge in High-shore	0,185	0%	3%	8%	89%	10%	10%	0%	28%
1230054	Fine-scale shoreline habitat units - High-shore intertidal zone	Low rock ledge in High-shore	0,069	5%	3%	45%	47%	0%	20%	na	41%
1230055	Fine-scale shoreline habitat units - High-shore intertidal zone	Mixture of High and Low rock ledge in High-shore	0,004	0%	0%	0%	100%	0%	20%	na	0%
1230056	Fine-scale shoreline habitat units - High-shore intertidal zone	Rock Broken Ledge High in High-shore	0,053	5%	0%	1%	94%	10%	10%	53%	0%
1230057	Fine-scale shoreline habitat units - High-shore intertidal zone	Rock Broken Ledge Low in High-shore	0,012	100%	0%	0%	0%	0%	20%	na	100%
1230059	Fine-scale shoreline habitat units - High-shore intertidal zone	Scattered rocks in High-shore	0,418	1%	1%	46%	52%	0%	20%	na	11%
1241049	Fine-scale shoreline habitat units - Mid-shore intertidal zone	Delagoa - Rock boulders in Mid-shore	0,108	0%	6%	94%	0%	20%	0%	0%	na
1241051	Fine-scale shoreline habitat units - Mid-shore intertidal zone	Delagoa - Broken rocks in Mid-shore	0,120	31%	3%	66%	0%	0%	20%	na	100%
1241053	Fine-scale shoreline habitat units - Mid-shore intertidal zone	Delagoa - High rock ledge in Mid-shore	0,150	2%	10%	88%	0%	20%	0%	9%	na
1241054	Fine-scale shoreline habitat units - Mid-shore intertidal zone	Delagoa - Low rock ledge in Mid-shore	0,439	30%	7%	63%	0%	0%	20%	na	100%
1241055	Fine-scale shoreline habitat units - Mid-shore intertidal zone	Delagoa - Mixture of High and Low rock ledge in Mid-shore	0,020	10%	0%	90%	0%	0%	20%	na	50%
1241056	Fine-scale shoreline habitat units - Mid-shore intertidal zone	Delagoa - Rock Broken Ledge High in Mid-shore	0,022	0%	0%	100%	0%	20%	0%	0%	na
1241057	Fine-scale shoreline habitat units - Mid-shore intertidal zone	Delagoa - Rock Broken Ledge Low in Mid-shore	0,030	0%	0%	100%	0%	0%	20%	na	0%
1241059	Fine-scale shoreline habitat units - Mid-shore intertidal zone	Delagoa - Scattered rocks in Mid-shore	0,180	48%	3%	49%	0%	0%	20%	na	100%
1242049	Fine-scale shoreline habitat units - Mid-shore intertidal zone	Natal - Solid Continuous Rocks in Mid-shore	0,568	0%	0%	29%	71%	20%	0%	0%	na
1242051	Fine-scale shoreline habitat units - Mid-shore intertidal zone	Natal - Broken rocks in Mid-shore	0,126	0%	0%	3%	97%	0%	20%	na	0%
1242053	Fine-scale shoreline habitat units - Mid-shore intertidal zone	Natal - High rock ledge in Mid-shore	0,477	0%	0%	4%	96%	20%	0%	0%	na
1242054	Fine-scale shoreline habitat units - Mid-shore intertidal zone	Natal - Low rock ledge in Mid-shore	0,208	0%	3%	5%	92%	0%	20%	na	13%

Code	Category of elements	Biodiversity elements	Surface (km sq.)	Area in MPA type A (%)	Area in MPA type B (%)	Area in MPA type C (%)	Area without protection (%)	A Target (%)	B Target (%)	% of A target achieved	% of B target achieved
1242055	Fine-scale shoreline habitat units - Mid-shore intertidal zone	Natal - Mixture of High and Low rock ledge in Mid-shore	0,015	0%	0%	0%	100%	0%	20%	na	0%
1242056	Fine-scale shoreline habitat units - Mid-shore intertidal zone	Natal - Rock Broken Ledge High in Mid-shore	0,041	0%	0%	0%	100%	20%	0%	0%	na
1242057	Fine-scale shoreline habitat units - Mid-shore intertidal zone	Natal - Rock Broken Ledge Low in Mid-shore	0,012	0%	0%	0%	100%	0%	20%	na	0%
1242059	Fine-scale shoreline habitat units - Mid-shore intertidal zone	Natal - Scattered rocks in Mid-shore	0,327	0%	0%	21%	79%	0%	20%	na	0%
1251049	Fine-scale shoreline habitat units - Low-shore intertidal zone	Delagoa - Rock boulders in Low-shore	0,041	4%	0%	96%	0%	20%	0%	20%	na
1251051	Fine-scale shoreline habitat units - Low-shore intertidal zone	Delagoa - Broken rocks in Low-shore	0,158	23%	27%	50%	0%	0%	20%	na	100%
1251053	Fine-scale shoreline habitat units - Low-shore intertidal zone	Delagoa - High rock ledge in Low-shore	0,328	24%	21%	55%	0%	20%	0%	100%	na
1251054	Fine-scale shoreline habitat units - Low-shore intertidal zone	Delagoa - Low rock ledge in Low-shore	0,670	54%	4%	42%	0%	0%	20%	na	100%
1251055	Fine-scale shoreline habitat units - Low-shore intertidal zone	Delagoa - Mixture of High and Low rock ledge in Low-shore	0,039	32%	23%	45%	0%	0%	20%	na	100%
1251056	Fine-scale shoreline habitat units - Low-shore intertidal zone	Delagoa - Rock Broken Ledge High in Low-shore	0,088	50%	23%	27%	0%	20%	0%	100%	na
1251057	Fine-scale shoreline habitat units - Low-shore intertidal zone	Delagoa - Rock Broken Ledge Low in Low-shore	0,127	44%	5%	51%	0%	0%	20%	na	100%
1251059	Fine-scale shoreline habitat units - Low-shore intertidal zone	Delagoa - Scattered rocks in Low-shore	0,536	19%	29%	52%	0%	0%	20%	na	100%
1251067	Fine-scale shoreline habitat units - Low-shore intertidal zone	Delagoa - Mixture of Broken low and High ledge in Low-shore	0,023	21%	31%	48%	0%	0%	20%	na	100%
1252049	Fine-scale shoreline habitat units - Low-shore intertidal zone	Natal - Rock boulders in Low-shore	0,247	0%	1%	13%	87%	20%	0%	0%	na
1252051	Fine-scale shoreline habitat units - Low-shore intertidal zone	Natal - Broken rocks in Low-shore	0,147	0%	4%	9%	87%	0%	20%	na	20%
1252053	Fine-scale shoreline habitat units - Low-shore intertidal zone	Natal - High rock ledge in Low-shore	0,985	0%	1%	11%	88%	20%	0%	0%	na
1252054	Fine-scale shoreline habitat units - Low-shore intertidal zone	Natal - Low rock ledge in Low-shore	0,445	0%	2%	15%	83%	0%	20%	na	11%
1252055	Fine-scale shoreline habitat units - Low-shore intertidal zone	Natal - Mixture of High and Low rock ledge in Low-shore	0,066	0%	0%	64%	36%	0%	20%	na	0%
1252056	Fine-scale shoreline habitat units - Low-shore intertidal zone	Natal - Rock Broken Ledge High in Low-shore	0,149	0%	2%	6%	92%	20%	0%	0%	na
1252057	Fine-scale shoreline habitat units - Low-shore intertidal zone	Natal - Rock Broken Ledge Low in Low-shore	0,012	0%	52%	0%	48%	0%	20%	na	100%

Code	Category of elements	Biodiversity elements	Surface (km sq.)	Area in MPA type A (%)	Area in MPA type B (%)	Area in MPA type C (%)	Area without protection (%)	A Target (%)	B Target (%)	% of A target achieved	% of B target achieved
1252059	Fine-scale shoreline habitat units - Low-shore intertidal zone	Natal - Scattered rocks in Low-shore	0,570	0%	3%	5%	92%	0%	20%	na	13%
1252060	Fine-scale shoreline habitat units - Low-shore intertidal zone	Natal - Solid Continuous Rocks in Low-shore	0,689	0%	0%	15%	85%	20%	0%	0%	na
1252067	Fine-scale shoreline habitat units - Low-shore intertidal zone	Natal - Mixture of Broken low and High ledge in Low-shore	0,019	0%	0%	0%	100%	0%	20%	na	0%
1260041	Fine-scale shoreline habitat units - Surf-zone	Mixture of Emergent and Submerged rocks in Surf-zone	1,431	4%	10%	11%	75%	0%	20%	na	68%
1260051	Fine-scale shoreline habitat units - Surf-zone	Broken rocks in Surf-zone	0,353	0%	2%	0%	98%	0%	20%	na	10%
1260052	Fine-scale shoreline habitat units - Surf-zone	Emergent rocks in Surf-zone	12,624	5%	7%	17%	71%	0%	10%	na	100%
1260054	Fine-scale shoreline habitat units - Surf-zone	Low rock ledge in Surf-zone	0,850	5%	1%	5%	89%	0%	20%	na	27%
1260055	Fine-scale shoreline habitat units - Surf-zone	Mixture of High and Low rock ledge in Surf-zone	0,025	25%	0%	0%	75%	0%	20%	na	100%
1260056	Fine-scale shoreline habitat units - Surf-zone	Rock Broken Ledge High in Surf-zone	0,132	0%	0%	0%	100%	10%	10%	0%	0%
1260057	Fine-scale shoreline habitat units - Surf-zone	Rock Broken Ledge Low in Surf-zone	0,041	7%	0%	23%	71%	0%	20%	na	34%
1260058	Fine-scale shoreline habitat units - Surf-zone	Submerged rocks in Surf-zone	3,564	15%	28%	16%	41%	0%	10%	na	100%
1260059	Fine-scale shoreline habitat units - Surf-zone	Scattered rocks in Surf-zone	1,284	0%	4%	5%	91%	0%	20%	na	21%
1260060	Fine-scale shoreline habitat units - Surf-zone	Solid Continuous Rocks in Surf-zone	0,305	0%	7%	3%	90%	10%	10%	0%	70%
1300101	Fine-scale shoreline habitat units - Sandy shore	Intermediate sandy shore	54,535	13%	12%	9%	66%	0%	20%	na	100%
1300104	Fine-scale shoreline habitat units - Sandy shore	Dissipative sandy shore	2,794	6%	0%	3%	91%	0%	20%	na	32%
1300103	Fine-scale shoreline habitat units - Sandy shore	Reflective sandy shore	12,507	10%	4%	2%	84%	0%	20%	na	73%
1300102	Fine-scale shoreline habitat units - Sandy shore	Intermediate sandy shore within 500m of an estuary	12,323	1%	13%	17%	68%	0%	20%	na	72%
1300105	Fine-scale shoreline habitat units - Sandy shore	Dissipative sandy shore within 500m of an estuary	0,616	4%	0%	4%	92%	0%	20%	na	22%
1300106	Fine-scale shoreline habitat units - Sandy shore	Reflective sandy shore within 500m of an estuary	0,734	4%	12%	7%	76%	0%	20%	na	84%
1912001	Estuaries Delagoa	Estuary - Kosi (open)	0,238	100%	0%	0%	0%	0%	20%	na	100%
1912076	Estuaries Delagoa	Estuary - Mgobozeleni (open)	0,177	0%	0%	100%	0%	0%	20%	na	0%

Code	Category of elements	Biodiversity elements	Surface (km sq.)	Area in MPA type A (%)	Area in MPA type B (%)	Area in MPA type C (%)	Area without protectio n (%)	A Target (%)	B Target (%)	% of A target achieved	% of B target achieved
1911002	Estuaries Natal	Estuary - St Lucia (open)	1,402	0%	0%	0%	100%	0%	20%	na	0%
1911005	Estuaries Natal	Estuary - Mlalazi (open)	0,037	0%	0%	0%	100%	0%	20%	na	0%
1911008	Estuaries Natal	Estuary - Mngeni (open)	2,942	0%	0%	0%	100%	0%	20%	na	0%
1911011	Estuaries Natal	Estuary - Mkomazi (open)	9,763	0%	0%	54%	46%	0%	20%	na	0%
1911012	Estuaries Natal	Estuary - Mzimkhulu (open)	15,996	0%	0%	0%	100%	0%	20%	na	0%
1921036	Estuaries Natal	Estuary - Mahlongwa (close)	0,166	0%	0%	100%	0%	0%	20%	na	0%
1921045	Estuaries Natal	Estuary - Sipingo (close)	0,274	0%	0%	0%	100%	0%	20%	na	0%
1921048	Estuaries Natal	Estuary - Mhlali (close)	0,260	0%	0%	0%	100%	0%	20%	na	0%
1921053	Estuaries Natal	Estuary - Zinkwazi (close)	0,139	0%	0%	0%	100%	0%	20%	na	0%
1921056	Estuaries Natal	Estuary - Thukela (close)	155,542	0%	0%	0%	100%	0%	20%	na	0%
1921058	Estuaries Natal	Estuary - Zotsha (close)	0,241	0%	0%	0%	100%	0%	20%	na	0%
1921060	Estuaries Natal	Estuary - Vungu (close)	0,147	0%	0%	0%	100%	0%	20%	na	0%
1921068	Estuaries Natal	Estuary - Mpenjati (close)	0,095	0%	0%	100%	0%	0%	20%	na	0%
1921072	Estuaries Natal	Estuary - Sandlundlu (close)	0,160	0%	0%	0%	100%	0%	20%	na	0%
1921073	Estuaries Natal	Estuary - Mtamvuna (close)	0,201	0%	0%	0%	100%	0%	20%	na	0%
2251191	Fish Adult Area (FAA)	Acanthopagrus vagus FAA	412,649	0%	1%	13%	87%	35%	0%	0%	na
2251103	Fish Nursery Area (FNA)	Acanthopagrus vagus FNA	280,337	8%	6%	16%	70%	40%	0%	21%	na
2251171	Fish Spawning Area (FSPA)	Acanthopagrus vagus FSPA	319,751	12%	6%	17%	66%	30%	0%	39%	na
2251229	Fish Distribution Area (FDA)	Anchichoerops natalensis FDA	24,023	0%	0%	16%	84%	25%	0%	0%	na
2251231	Fish Distribution Area (FDA)	Anthias connelli FDA	98,398	8%	4%	16%	72%	35%	0%	22%	na
2251175	Fish Distribution Area (FDA)	Apolemichthys kingi FDA	1218,129	6%	4%	9%	81%	25%	0%	24%	na
2251188	Fish Adult Area (FAA)	Argyrops spinifer FAA	558,487	6%	5%	14%	75%	25%	0%	25%	na
2251132	Fish Nursery Area (FNA)	Argyrops spinifer FNA	774,136	0%	0%	5%	94%	25%	0%	0%	na
2251128	Fish Adult Area (FAA)	Argyrosomus japonicus FAA	1065,443	6%	4%	9%	82%	25%	0%	23%	na
2251129	Fish migration Pathway to Nursery Area (FPNA)	Argyrosomus japonicus FPNA	356,000	0%	0%	9%	91%	35%	0%	0%	na
2251155	Fish migration Pathway to Spawning Area (FPSPA)	Argyrosomus japonicus FPSPA	69,764	0%	0%	17%	83%	32%	0%	0%	na

Code	Category of elements	Biodiversity elements	Surface (km sq.)	Area in MPA type A (%)	Area in MPA type B (%)	Area in MPA type C (%)	Area without protectio n (%)	A Target (%)	B Target (%)	% of A target achieved	% of B target achieved
2251312	Fish Spawning Area (FSPA)	Argyrosomus japonicus FSPA	203,092	8%	7%	11%	74%	35%	0%	24%	na
2251122	Fish Adult Area (FAA)	Argyrosomus thorpei FAA	962,062	4%	4%	10%	82%	30%	0%	15%	na
2251135	Fish Nursery Area (FNA)	Argyrosomus thorpei FNA	1169,743	4%	3%	9%	84%	25%	0%	15%	na
2251136	Fish Spawning Area (FSPA)	Argyrosomus thorpei FSPA	668,936	7%	5%	14%	73%	35%	0%	20%	na
2251100	Fish Adult Area (FAA)	Atractoscion aequidens FAA	903,674	7%	5%	11%	77%	35%	0%	21%	na
2251107	Fish migration Pathway to Nursery Area (FPNA)	Atractoscion aequidens FPNA	937,953	5%	4%	11%	81%	35%	0%	13%	na
2251108	Fish migration Pathway to Spawning Area (FPSPA)	Atractoscion aequidens FPSPA	1290,478	6%	4%	9%	81%	30%	0%	19%	na
2251101	Fish Spawning Area (FSPA)	Atractoscion aequidens FSPA	1245,821	4%	3%	9%	83%	30%	0%	13%	na
2251111	Fish Distribution Area (FDA)	Caffrogobius caffer FDA	873,842	8%	5%	6%	81%	20%	0%	39%	na
2251163	Fish Distribution Area (FDA)	Caffrogobius natalensis FDA	7865,137	3%	2%	4%	91%	25%	0%	11%	na
2251079	Fish Distribution Area (FDA)	Chaetodon marleyi FDA	576,791	9%	6%	15%	70%	30%	0%	31%	na
2251071	Fish Distribution Area (FDA)	Chirodactylus jessicalenorum FDA	8762,437	3%	2%	4%	91%	0%	25%	na	20%
2251053	Fish Distribution Area (FDA)	Chrysobelphus lophus FDA	17,220	25%	0%	63%	12%	42%	0%	60%	na
2251207	Fish Distribution Area (FDA)	Chrysoblephus anglicus FDA	266,744	6%	6%	13%	76%	25%	0%	23%	na
2251306	Fish Distribution Area (FDA)	Chrysoblephus cristiceps FDA	1047,295	4%	4%	10%	82%	30%	0%	14%	na
2251307	Fish Adult Area (FAA)	Chrysoblephus puniceus FAA	674,511	11%	7%	8%	75%	30%	0%	35%	na
2251123	Fish Nursery Area (FNA)	Chrysoblephus puniceus FNA	932,367	5%	4%	9%	83%	40%	0%	13%	na
2251298	Fish Spawning Area (FSPA)	Chrysoblephus puniceus FSPA	266,744	6%	6%	13%	76%	30%	0%	19%	na
2251157	Fish Adult Area (FAA)	Cymatoceps nasutus FAA	268,430	6%	6%	13%	76%	25%	0%	25%	na
2251176	Fish Nursery Area (FNA)	Cymatoceps nasutus FNA	109,353	0%	2%	33%	65%	25%	0%	0%	na
2251178	Fish Spawning Area (FSPA)	Cymatoceps nasutus FSPA	817,304	5%	4%	10%	81%	20%	0%	25%	na
2251139	Fish Distribution Area (FDA)	Dichistius capensis FDA	322,468	7%	6%	14%	73%	35%	0%	20%	na
2251226	Fish Distribution Area (FDA)	Dichistius multifasciatus FDA	143,217	4%	3%	15%	77%	0%	35%	na	22%
2251142	Fish Distribution Area (FDA)	Dinoperca petersi FDA	588,690	9%	5%	14%	71%	25%	0%	38%	na
2251143	Fish Distribution Area (FDA)	Diplodus cervinus hottentotus FDA	817,411	3%	4%	7%	86%	25%	0%	11%	na
2251144	Fish Distribution Area (FDA)	Diplodus sargus capensis FDA	1226,102	4%	3%	9%	84%	25%	0%	14%	na

Code	Category of elements	Biodiversity elements	Surface (km sq.)	Area in MPA type A (%)	Area in MPA type B (%)	Area in MPA type C (%)	Area without protection (%)	A Target (%)	B Target (%)	% of A target achieved	% of B target achieved
2251308	Fish Distribution Area (FDA)	Epinephelus albomarginatus FDA	694,458	7%	5%	6%	81%	20%	0%	37%	na
2251146	Fish Distribution Area (FDA)	Epinephelus andersoni FDA	576,317	8%	5%	7%	79%	25%	0%	33%	na
2251257	Fish Distribution Area (FDA)	Epinephelus lanceolatus FDA	98,398	8%	4%	16%	72%	25%	0%	31%	na
2251151	Fish Distribution Area (FDA)	Epinephelus marginatus FDA	1125,860	5%	4%	11%	80%	25%	0%	20%	na
2251259	Fish Distribution Area (FDA)	Epinephelus tukula FDA	98,398	8%	4%	16%	72%	25%	0%	31%	na
2251260	Fish Distribution Area (FDA)	Gerres methueni FDA	98,398	8%	4%	16%	72%	40%	0%	19%	na
2251292	Fish Distribution Area (FDA)	Hippocampus whitei FDA	314,434	0%	0%	0%	100%	40%	0%	0%	na
2251169	Fish Distribution Area (FDA)	Hyporhamphus capensis FDA	481,030	7%	5%	15%	72%	20%	0%	36%	na
2254124	Fish Distribution Area (FDA)	Latimeria chalumnae FDA	0,023	0%	0%	0%	100%	25%	0%	0%	na
2254125	Fish Adult Area (FAA)	Lichia amia FAA	9535,612	2%	2%	3%	93%	20%	0%	9%	na
2254165	Fish migration Pathway to Nursery Area (FPNA)	Lichia amia FPNA	1247,440	6%	4%	10%	80%	30%	0%	20%	na
2254166	Fish migration Pathway to Spawning Area (FPSPA)	Lichia amia FPSPA	2344,450	4%	3%	9%	83%	32%	0%	14%	na
2254167	Fish Spawning Area (FSPA)	Lichia amia FSPA	7865,126	3%	2%	4%	91%	16%	16%	18%	13%
2254133	Fish Distribution Area (FDA)	Liza tricuspidens FDA	1253,664	6%	5%	11%	78%	25%	0%	25%	na
2254134	Fish Distribution Area (FDA)	Lutjanus argentimaculatus FDA	1125,394	4%	3%	9%	84%	35%	0%	11%	na
2254182	Fish Distribution Area (FDA)	Lutjanus rivulatus FDA	3678,177	0%	0%	3%	97%	0%	20%	na	0%
2254299	Fish Distribution Area (FDA)	Lutjanus sanguineus FDA	2341,034	4%	3%	9%	83%	30%	0%	15%	na
2254138	Fish Distribution Area (FDA)	Myxus capensis FDA	880,787	2%	2%	10%	85%	35%	0%	7%	na
2254141	Fish Distribution Area (FDA)	Neoscorpis lithophilus FDA	899,236	0%	0%	7%	93%	25%	0%	1%	na
2254145	Fish Distribution Area (FDA)	Oplegnathus conwayi FDA	984,748	3%	3%	7%	87%	35%	0%	8%	na
2254117	Fish Distribution Area (FDA)	Oplegnathus robinsoni FDA	2344,450	4%	3%	9%	83%	20%	0%	22%	na
2254120	Fish Adult Area (FAA)	Otolithes ruber FAA	1026,385	4%	3%	9%	84%	20%	0%	19%	na
2254116	Fish Nursery Area (FNA)	Otolithes ruber FNA	7491,297	3%	2%	4%	91%	0%	20%	na	25%
2254150	Fish Spawning Area (FSPA)	Otolithes ruber FSPA	1226,102	4%	3%	9%	84%	20%	0%	18%	na
2254152	Fish Adult Area (FAA)	Pachymetopon aeneum FAA	1979,086	5%	4%	9%	83%	20%	0%	23%	na
2254313	Fish Nursery Area (FNA)	Pachymetopon aeneum FNA	9877,602	3%	2%	4%	91%	0%	20%	na	24%

Code	Category of elements	Biodiversity elements	Surface (km sq.)	Area in MPA type A (%)	Area in MPA type B (%)	Area in MPA type C (%)	Area without protection (%)	A Target (%)	B Target (%)	% of A target achieved	% of B target achieved
2254272	Fish Spawning Area (FSPA)	Pachymetopon aeneum FSPA	1134,923	4%	3%	9%	83%	0%	20%	na	37%
2252124	Fish Distribution Area (FDA)	Pachymetopon grande FDA	271,856	6%	5%	13%	76%	25%	0%	25%	na
2252165	Fish Distribution Area (FDA)	Pavoclinus mentalis FDA	720,786	3%	3%	6%	88%	20%	0%	17%	na
2252166	Fish Adult Area (FAA)	Petrus rupestris FAA	2344,450	4%	3%	9%	83%	22%	0%	20%	na
2252167	Fish migration Pathway to Nursery Area (FPNA)	Petrus rupestris FPNA	7273,499	0%	0%	2%	98%	11%	11%	0%	0%
2252133	Fish migration Pathway to Spawning Area (FPSPA)	Petrus rupestris FPSPA	178,350	37%	22%	27%	13%	25%	0%	100%	na
2252134	Fish Spawning Area (FSPA)	Petrus rupestris FSPA	1123,014	4%	3%	9%	84%	25%	0%	15%	na
2252182	Fish Distribution Area (FDA)	Polyamblyodon germanum FDA	2633,054	0%	0%	0%	100%	0%	20%	na	0%
2252299	Fish Distribution Area (FDA)	Polysteganus coeruleopunctatus FDA	334,955	0%	0%	0%	100%	30%	0%	0%	na
2252138	Fish Distribution Area (FDA)	Polysteganus praeorbitalis FDA	354,440	0%	1%	15%	84%	25%	0%	0%	na
2252141	Fish Adult Area (FAA)	Polysteganus undulosus FAA	288,029	0%	1%	14%	85%	25%	0%	0%	na
2252145	Fish migration Pathway to Nursery Area (FPNA)	Polysteganus undulosus FPNA	292,166	0%	1%	14%	85%	25%	0%	0%	na
2252117	Fish migration Pathway to Spawning Area (FPSPA)	Polysteganus undulosus FPSPA	263,985	6%	5%	13%	76%	20%	0%	30%	na
2252120	Fish Spawning Area (FSPA)	Polysteganus undulosus FSPA	1026,385	4%	3%	9%	84%	20%	0%	19%	na
2252116	Fish Adult Area (FAA)	Pomadasys commersonnii FAA	3566,222	3%	2%	3%	92%	0%	20%	na	23%
2252149	Fish Nursery Area (FNA)	Pomadasys commersonnii FNA	3675,766	0%	0%	3%	96%	25%	0%	0%	na
2252150	Fish Spawning Area (FSPA)	Pomadasys commersonnii FSPA	4037,852	4%	3%	6%	88%	20%	0%	18%	na
2252060	Fish Distribution Area (FDA)	Pomadasys furcatum FDA	10108,932	0%	0%	1%	99%	0%	20%	na	0%
2252152	Fish Adult Area (FAA)	Pomadasys olivaceum FAA	1979,086	5%	4%	9%	83%	20%	0%	23%	na
2252313	Fish Nursery Area (FNA)	Pomadasys olivaceum FNA	9877,602	3%	2%	4%	91%	0%	20%	na	24%
2253124	Fish Spawning Area (FSPA)	Pomadasys olivaceum FSPA	0,023	0%	0%	0%	100%	25%	0%	0%	na
2253125	Fish Adult Area (FAA)	Pomatomus saltatrix FAA	2242,647	4%	3%	8%	84%	30%	0%	14%	na
2253166	Fish migration Pathway to Nursery Area (FPNA)	Pomatomus saltatrix FPNA	2374,262	0%	0%	0%	100%	22%	0%	0%	na
2253133	Fish migration Pathway to Spawning Area (FPSPA)	Pomatomus saltatrix FPSPA	627,028	7%	5%	11%	77%	25%	0%	27%	na
2253134	Fish Spawning Area (FSPA)	Pomatomus saltatrix FSPA	80,534	4%	6%	17%	73%	35%	0%	13%	na

Code	Category of elements	Biodiversity elements	Surface (km sq.)	Area in MPA type A (%)	Area in MPA type B (%)	Area in MPA type C (%)	Area without protection (%)	A Target (%)	B Target (%)	% of A target achieved	% of B target achieved
2253299	Fish Distribution Area (FDA)	Porcostoma dentata FDA	1405,259	0%	0%	0%	100%	30%	0%	0%	na
2253138	Fish Distribution Area (FDA)	Redigobius dewaalii FDA	160,820	9%	5%	12%	74%	25%	0%	35%	na
2253117	Fish migration Pathway to Nursery Area (FPNA)	Rhabdosargus holubi FPNA	4041,248	4%	3%	6%	88%	20%	0%	18%	na
2253120	Fish migration Pathway to Spawning Area (FPSPA)	Rhabdosargus holubi FPSPA	0,023	0%	0%	0%	100%	20%	0%	0%	na
2253150	Fish Spawning Area (FSPA)	Rhabdosargus holubi FSPA	0,023	0%	0%	0%	100%	20%	0%	0%	na
2253152	Fish Adult Area (FAA)	Rhabdosargus sarba FAA	0,023	0%	0%	0%	100%	20%	0%	0%	na
3454272	Fish Nursery Area (FNA)	Rhabdosargus sarba FNA	3681,639	2%	1%	4%	92%	0%	20%	na	18%
3452165	Fish Spawning Area (FSPA)	Rhabdosargus sarba FSPA	3136,773	4%	3%	7%	86%	20%	0%	19%	na
3452167	Fish Distribution Area (FDA)	Rhabdosargus thorpei FDA	2777,472	0%	0%	4%	96%	10%	10%	0%	1%
3452182	Fish migration Pathway to Nursery Area (FPNA)	Sardinops sagax FPNA	1491,234	0%	0%	2%	98%	0%	20%	na	0%
3452141	Fish migration Pathway to Spawning Area (FPSPA)	Sardinops sagax FPSPA	983,713	0%	0%	2%	98%	20%	0%	0%	na
3452145	Fish Spawning Area (FSPA)	Sardinops sagax FSPA	1298,467	0%	0%	7%	93%	20%	0%	0%	na
3452116	Fish Adult Area (FAA)	Sarpa salpa FAA	2397,198	3%	3%	7%	87%	0%	20%	na	30%
3452149	Fish Nursery Area (FNA)	Sarpa salpa FNA	3052,714	2%	2%	5%	91%	20%	0%	9%	na
3452060	Fish Spawning Area (FSPA)	Sarpa salpa FSPA	0,000	0%	0%	4%	96%	0%	20%	na	0%
3452313	Fish Adult Area (FAA)	Scomber japonicus FAA	258,509	0%	1%	33%	66%	0%	20%	na	4%
3452272	Fish migration Pathway to Nursery Area (FPNA)	Scomber japonicus FPNA	3681,639	2%	1%	4%	92%	0%	20%	na	18%
3453165	Fish migration Pathway to Spawning Area (FPSPA)	Scomber japonicus FPSPA	7453,450	2%	2%	4%	92%	20%	0%	11%	na
3453167	Fish Spawning Area (FSPA)	Scomber japonicus FSPA	8863,095	0%	0%	1%	99%	10%	10%	0%	0%
3453182	Fish Adult Area (FAA)	Scomberomorus commerson FAA	7520,827	0%	0%	2%	98%	0%	20%	na	0%
3453141	Fish migration Pathway to Adult Area (FPAA)	Scomberomorus commerson FPAA	983,713	0%	0%	2%	98%	20%	0%	0%	na
3453145	Fish migration Pathway to Spawning Area (FPSPA)	Scomberomorus commerson FPSPA	1298,467	0%	0%	7%	93%	20%	0%	0%	na
3453116	Fish Distribution Area (FDA)	Taeniodes esquivel FDA	8355,898	2%	2%	3%	92%	0%	20%	na	21%
3453149	Fish Distribution Area (FDA)	Taeniodes jacksoni FDA	6767,518	2%	2%	4%	92%	20%	0%	12%	na

Code	Category of elements	Biodiversity elements	Surface (km sq.)	Area in MPA type A (%)	Area in MPA type B (%)	Area in MPA type C (%)	Area without protection (%)	A Target (%)	B Target (%)	% of A target achieved	% of B target achieved
3453060	Fish Distribution Area (FDA)	Torquigener marleyi FDA	9061,594	0%	0%	2%	98%	0%	20%	na	0%
3453313	Fish Distribution Area (FDA)	Umbrina robinsoni FDA	483,591	0%	0%	22%	77%	0%	20%	na	2%
2251310	Shark Distribution Area (SDA)	Anacanthobatis marmoratus SDA	823,290	1%	3%	4%	92%	40%	0%	2%	na
2251024	Shark Adult Area (SAA)	Carcharhinus leucas SAA	1132,824	0%	0%	5%	94%	0%	30%	na	1%
2251034	Shark Nursery Area (SNA)	Carcharhinus leucas SNA	3126,010	1%	3%	3%	94%	35%	0%	2%	na
2251029	Shark migration Pathway to Adult Area (SPAA)	Carcharhinus leucas SPAA	876,581	6%	4%	10%	80%	20%	0%	30%	na
2251030	Shark migration Pathway to Nursery Area (SPNA)	Carcharhinus leucas SPNA	876,581	6%	4%	10%	80%	20%	0%	30%	na
2251039	Shark migration Pathway to Spawning Area (SPSPA)	Carcharhinus leucas SPSPA	876,573	6%	4%	10%	80%	40%	0%	15%	na
2251008	Shark Adult Area (SAA)	Carcharhinus obscurus SAA	1424,659	13%	13%	18%	55%	0%	30%	na	88%
2251017	Shark Nursery Area (SNA)	Carcharhinus obscurus SNA	724,233	0%	0%	8%	91%	45%	0%	0%	na
2251022	Shark migration Pathway to Adult Area (SPAA)	Carcharhinus obscurus SPAA	852,432	7%	4%	10%	78%	0%	30%	na	38%
2251051	Shark migration Pathway to Spawning Area (SPSPA)	Carcharhinus obscurus SPSPA	4037,852	4%	3%	6%	88%	30%	0%	12%	na
2254010	Shark Adult Area (SAA)	Carcharias taurus SAA	8288,377	3%	2%	4%	91%	15%	15%	19%	14%
2254311	Shark migration Pathway to Adult Area (SPAA)	Carcharias taurus SPAA	2424,835	0%	2%	2%	95%	15%	15%	3%	16%
2254027	Shark migration Pathway to Nursery Area (SPNA)	Carcharias taurus SPNA	8677,525	3%	2%	4%	91%	20%	0%	14%	na
2254040	Shark migration Pathway to Spawning Area (SPSPA)	Carcharias taurus SPSPA	5201,076	0%	0%	2%	98%	22%	0%	0%	na
2252027	Shark Mating Area (SSPA)	Carcharias taurus SSPA	1292,054	18%	14%	18%	49%	20%	0%	92%	na
2252040	Shark Distribution Area (SDA)	Carcharodon carcharias SDA	3242,484	0%	0%	4%	96%	22%	0%	0%	na
2253010	Shark Distribution Area (SDA)	Dipturus campbelli SDA	0,020	0%	0%	0%	100%	20%	20%	0%	0%
2253311	Shark Distribution Area (SDA)	Pristis microdon SDA	868,340	0%	0%	14%	86%	10%	10%	0%	2%
2253040	Shark Distribution Area (SDA)	Pristis zijsron SDA	3242,484	0%	0%	4%	96%	22%	0%	0%	na
3454010	Shark Distribution Area (SDA)	Rhina ancylostoma SDA	3681,639	2%	1%	4%	92%	10%	10%	22%	14%
3454311	Shark Adult Area (SAA)	Rhinobatos annulatus SAA	10660,248	2%	2%	4%	91%	10%	10%	24%	23%
3454027	Shark Nursery Area (SNA)	Rhinobatos annulatus SNA	3681,639	2%	1%	4%	92%	20%	0%	11%	na

Code	Category of elements	Biodiversity elements	Surface (km sq.)	Area in MPA type A (%)	Area in MPA type B (%)	Area in MPA type C (%)	Area without protection (%)	A Target (%)	B Target (%)	% of A target achieved	% of B target achieved
3452010	Shark Mating Area (SSPA)	Rhinobatos annulatus SSPA	3681,639	2%	1%	4%	92%	10%	10%	22%	14%
3452311	Shark Distribution Area (SDA)	Rhinocodon typus SDA	3315,018	2%	2%	6%	90%	10%	10%	24%	22%
3452027	Shark Distribution Area (SDA)	Scylliogaleus quecketti SDA	0,000	2%	1%	4%	92%	20%	0%	11%	na
3453010	Shark Distribution Area (SDA)	Sphyrna mokarran SDA	9904,229	2%	2%	4%	92%	10%	10%	24%	18%
3453027	Shark Distribution Area (SDA)	Urogymnus asperrimus SDA	9904,229	2%	2%	4%	92%	20%	0%	12%	na
2230002	Turtles nesting sites	Leather backs Nesting Site	17,379	27%	47%	26%	0%	20%	60%	100%	90%
2230001	Turtles nesting sites	Logger heads Nesting Site	17,379	27%	47%	26%	0%	20%	60%	100%	90%
2245001	Ceataceans Distribution Area (MDA)	Bottle nose Dolphin MDA	1640,645	5%	4%	9%	82%	15%	15%	31%	25%
2245002	Ceataceans Distribution Area (MDA)	Hump back Dolphin MDA	865,535	6%	4%	10%	80%	20%	20%	30%	21%
2246002	Ceataceans Distribution Area (MDA)	Hump back whales MDA	8751,424	3%	2%	4%	91%	15%	15%	18%	14%
2246001	Ceataceans Distribution Area (MDA)	Sperm whales MDA	211412,139	0%	0%	0%	100%	15%	15%	0%	0%
3446002	Ceataceans Migration Pathway (MMP)	Hump back whale MMP	2033,265	8%	8%	13%	71%	20%	0%	41%	na
